# Characterizing the diversity of MHC conserved extended haplotypes using families from the United Arab Emirates

**DOI:** 10.1038/s41598-022-11256-y

**Published:** 2022-05-03

**Authors:** Halima Alnaqbi, Guan K. Tay, Sarah El Hajj Chehadeh, Habiba Alsafar

**Affiliations:** 1 Center for Biotechnology, Khalifa University of Science and Technology, P.O. BOX 127788, Abu Dhabi, UAE; 2 Department of Biomedical Engineering, Khalifa University of Science and Technology, Abu Dhabi, United Arab Emirates; 3 Division of Psychiatry, UWA Medical School, The University of Western Australia, Perth, WA Australia; 4 School of Medical and Health Sciences, Edith Cowan University, Joondalup, WA Australia; 5 Department of Pharmacology, College of Medicine and Health Sciences, Khalifa University of Science and Technology, Abu Dhabi, United Arab Emirates; 6 Department of Genetics and Molecular Biology, College of Medicine and Health Sciences, Khalifa University of Science and Technology, Abu Dhabi, United Arab Emirates

**Keywords:** Immunogenetics, Genetics, Haplotypes, Sequencing, Evolutionary genetics

## Abstract

Aside from its anthropological relevance, the characterization of the allele frequencies of genes in the human Major Histocompatibility Complex (MHC) and the combination of these alleles that make up MHC conserved extended haplotypes (CEHs) is necessary for histocompatibility matching in transplantation as well as mapping disease association loci. The structure and content of the MHC region in Middle Eastern populations remain poorly characterized, posing challenges when establishing disease association studies in ethnic groups that inhabit the region and reducing the capacity to translate genetic research into clinical practice. This study was conceived to address a gap of knowledge, aiming to characterize CEHs in the United Arab Emirates (UAE) population through segregation analysis of high-resolution, pedigree-phased, MHC haplotypes derived from 41 families. Twenty per cent (20.5%) of the total haplotype pool derived from this study cohort were identified as putative CEHs in the UAE population. These consisted of CEHs that have been previously detected in other ethnic groups, including the South Asian CEH 8.2 [HLA- C*07:02-B*08:01-DRB1*03:01-DQA1*05:01-DQB1*02:01 (H.F. 0.094)] and the common East Asian CEH 58.1 [HLA- C*03:02-B*58:01-DRB1*03:01- DQA1*05:01-DQB1*02:01 (H.F. 0.024)]. Additionally, three novel CEHs were identified in the current cohort, including HLA- C*15:02-B*40:06-DRB1*16:02-DQB1*05:02 (H.F. 0.035), HLA- C*16:02-B*51:01-DRB1*16:01-DQA1*01:02-DQB1*05:02 (H.F. 0.029), and HLA- C*03:02-B*58:01-DRB1*16:01-DQA1*01:02-DQB1*05:02 (H.F. 0.024). Overall, the results indicate a substantial gene flow with neighbouring ethnic groups in the contemporary UAE population including South Asian, East Asian, African, and European populations. Importantly, alleles and haplotypes that have been previously associated with autoimmune diseases (e.g., Type 1 Diabetes) were also present. In this regard, this study emphasizes that an appreciation for ethnic differences can provide insights into subpopulation-specific disease-related polymorphisms, which has remained a difficult endeavour.

## Introduction

Interest in the genes of the Major Histocompatibility Complex (MHC), and in particular the Human Leukocyte Antigens (HLA), on the short arm of chromosome 6 is primarily due to their involvement in determining the histocompatibility between organs or cells for transplantation purposes. There are over 300 genes in this short 3 to 5 megabase region, many of which are highly polymorphic, and many belong to multigene families (HLA, C4, TAP, Cyp21, LMP, and others)^1–3^. The HLA class I (HLA-A, HLA-C, HLA-B) and HLA class II (HLA-DR, HLA-DQ, HLA-DP) genes located within this region encode important proteins for cell surface antigen presentation and are key components of the immune system, hence their involvement processes that might lead to autoimmune diseases^2,4–6^.

The high degree of molecular polymorphism observed in the classical HLA class I and class II genes reflect their direct involvement as antigen-presenting molecules against the variety of pathogens encountered throughout human evolution^7,8^. As a result of DNA insertion, deletion, and gene duplication events, distances between MHC loci, and hence the size of MHC haplotypes, may vary between different individuals^2^. While allelic and haplotype frequencies are relatively stable within an ethnic group and often definable by the Hardy–Weinberg equilibrium, these frequencies may vary substantially across populations^7^.

Arguably, the first description of conserved DNA blocks within the MHC was the combination of complement genes Bf-C2-C4A-C4B, by Alper et al. in 1983^9^. Upon coining the term MHC ‘complotype’ for the sub-region of the entire region between HLA-B (the centromeric end of the class I region) and HLA-DR (the telomeric end of the class II region); the group has continued to describe long-range or extended conservation within MHC haplotypes^10–12^. Subsequently, several groups have supported this hypothesis by reporting that unrelated individuals from well-defined human populations share short blocks of conserved DNA sequence having precise HLA allele combinations of two or more neighbouring loci within the MHC region^13–16^. On the other hand, far longer fragments of common conserved MHC DNA sequences occur in people from the same population or ancestry^8,15^. Those long fragments consist of combinations of four or more HLA loci and were termed ‘Conserved Extended Haplotypes (CEHs)’ by Alper et al.^12,17^, supratypes^18^ and ‘Ancestral Haplotypes (AHs)’ by the Dawkins group in Australia^3,19^. Both CEH and AH are commonly used. For consistency, the term ‘CEH’ will be used throughout this report to refer to conserved, long stretches of DNA that span more than 2.7 megabases (Mb) and extend from HLA-C to HLA-DQB1^8^. The extent of the CEHs has since been expanded to include the region telomeric of HLA-A, at least as far as the microsatellite marker D6S105^20^. A haplotype must have a minimum required frequency of 0.005 in a certain population to be considered a common CEH. Nonetheless, the minimum CEH frequency cutoff should also be dependent on the sample size such that a study with a small sample size requires the use of a higher frequency than a study with larger sample size.

According to Dawkins and Lloyd ^21^, MHC CEHs have been carried by different ancestral groups which have migrated out of Africa^21^. As a result of ethnic admixture, new MHC haplotypes have emerged and gradually become fixed in human populations and been perpetuated^14,19^. This has, in part, given rise to the unique population-specific frequencies which are now observed.

Some CEHs are ethnic-specific and may have arisen from specific combinations of connected blocks or the ancestral sequences^21^. Subsequently, the HLA markers included inside a specific block would predictably be similar, or nearly identical, among unrelated people^8^. In this context, MHC CEHs have been used to characterize human diversity, and ethnic origin, or to identify and localize disease susceptibility genes, especially those related to autoimmune diseases^4–6,18^ and for transplant matching^22,23^.

The characterization of the genetic architecture of any population is useful prior to conducting genetic association studies^4^. MHC disease association studies have been dominated by analyses based on populations of European ancestries. However, this is gradually changing, allowing researchers to fill the knowledge gaps in disease risk predictions in some ethnic groups^24^. Nevertheless, despite the efforts of the Haplotype Map (HapMap) project and other international consortia^25^, the genome structure, including that of the MHC, of populations from the Middle East remains poorly characterized, calling for the need to encourage disease association studies in the region as highlighted in our recent review^26^. The distribution of ancestry category of Genome-Wide Association Studies (GWAS) retrieved in 2019, showed that studies on Greater Middle Eastern/ Native American/ Oceanian altogether represent only 1.24% of the total studies^24,27^.

The knowledge gap in the Arabian genome influences the ability of healthcare in the region to translate research outcomes from genetic studies into clinical practice, especially for critical clinical assays such as histocompatibility matching. In this regard, more effort should be put into studying the MHC region of the Arabian population, particularly for individuals of Arabian ancestry, to offer better healthcare and benefit from the new paradigm of healthcare referred to as personalized or precision medicine.

Genotypically MHC-identical individuals can be found among siblings from a nuclear family^28^, and haplotypes are definable by segregation studies of the MHC genes carried in families^4^. Henceforth, family segregation analyses remain the gold standard for defining the structure of MHC CEHs and are preferred over population data which is less reliable due to the reliance on bioinformatics algorithms that infer linkage between loci.

There have been notable efforts in characterizing the MHC region and HLA genes in populations of the Arabian Peninsula from Bahrain^29^, Kuwait^30^, and Saudi Arabia^31,32^ to overcome the knowledge gap. By combining high-resolution typing by next-generation sequencing (NGS) with haplotype segregation analysis of family pedigrees, this report adds to these efforts by presenting data based on a powerful strategy. Although often grouped for their shared language, history, and culture, the populations of the Arabian Peninsula represent a genetically diverse group. The United Arab Emirates (UAE) is situated in the southeast of the Arabian Peninsula, an ethnically diverse region that has emerged as a result of social and cultural influences arising from important bidirectional human migration events between the African, European, and Asian continents. The original people of the Arabian Peninsula lived a nomadic lifestyle, migrating around the peninsula in search of suitable waterholes, creating settlements that served as a hub for commerce and cultural exchange. The subsequent establishment of trade routes^33^ has enhanced bidirectional gene flow into and out of the area^34^, resulting in the present diversity of contemporary Arabia. This study characterizes and identifies conserved HLA CEHs of the UAE populations using high-resolution HLA pedigree-phased haplotypes. With the UAE recently establishing its national organ registry program, this study provides insights on the MHC of the UAE population, which is important for matching recipients to appropriate donors. In time, our understanding of the involvement of specific alleles of relevant MHC genes in autoimmune disease is expected to be revealed.

## Methods

### Recruitment

Families were approached and briefed on the study and invited to participate. The cohort also included a subset of five families that have been previously published by Tay, et al. ^35^. Those families included healthy parents and at least one child with Type 1 Diabetes. Specifically, only the phased haplotypes of the healthy parents were retained for the current study. Families were randomly recruited from different parts of the UAE including northern, western, eastern, and south-eastern regions. All the participants recruited for the study were UAE nationals. Nonetheless, no sub-ethnic or country of ancestral origin information was collected from the recruited participants.

### Ethics declarations

All participants who chose to participate in the study completed a consent form and a questionnaire approved by Mafraq Hospital’s Institutional Review Board (IRB) committee (MAF-REC 07/2016 04) and Dubai Health Authority (DSREC-07/2020_39). Informed written consent was obtained from all the participants, and they authorized the storage of their DNA samples. Written informed consent was obtained from the parent of participants under the age of 18 years at the time of sample collection. All methods were carried out in accordance with relevant guidelines and regulations approved jointly by the IRB committee at Mafraq Hospital (MAF-REC 07/2016 04) and Dubai Health Authority (DSREC-07/2020_39).

### Sample collection and DNA extraction

In total, 235 saliva samples were collected from 41 UAE families, including one 3-generations family (family ID: HF8), using the Oragene-DNA collection kit (Genotek, Ottawa, Canada) according to the guidelines provided by the manufacturer. Genomic DNA (gDNA) was extracted from buccal cells using prepIT L2P reagents supplied with the Oragene-DNA kit (DNA Genotek, Canada), as per the manufacturer’s instructions. The quality of the gDNA was verified by OD260/OD280 > 1.8 measurements performed on Nanodrop One UV–Vis Spectrophotometer (Thermo Fisher Scientific, Waltham, USA) and by agarose gel. The concentration of each gDNA sample was measured using the dsDNA broad range fluorescence-based quantitation method (Denovix, Wilmington, USA).

### High-resolution HLA typing by NGS

High-resolution HLA typing was conducted using the Holotype HLA 96/11 library kit (Omixon, Budapest, Hungary) according to the manufacturer’s protocol. The Holotype HLA 96/11 kit uses long-range PCR amplification in the gDNA sample preparation step to provide comprehensive gene coverage for up to 11 HLA loci (HLA-A, HLA-B, HLA-C, HLA-DRB1/3/4/5, HLA-DQA1, HLA-DQB1, HLA-DPA1, HLA-DPB1). The library preparation step includes enzymatic fragmentation, end-repair, and ligation with indexed adaptors for each individual sample. The libraries are then combined into a single pooled library and size-selected using AMPure XP beads (Beckman Coulter, Massachusetts, USA). The concentration of the final library is determined using KAPPA library quantification ROX low kit on the Viaa7 Real-time PCR instrument (Applied Biosystems, Foster City, USA) (Kappa Biosystems, Wilmington, USA). The final library is then loaded onto the Illumina Miseq platform (Illumina, San Diego, USA). For analyses of results, FASTQ sequencing files are imported into Omixon’s HLA Twin Software v4.2.0 (Budapest, Hungary) where sequences are aligned to the most updated version of the International ImMunoGeneTics/ HLA (IMGT/HLA) database (www.ebi.ac.uk/imgt/hla/) using two independent computational algorithms for high confidence allele calling.

### Segregation analysis

Segregation analysis by pedigree was independently conducted by the co-authors, and all haplotypes assigned by these individuals were concordant. Each family had identical 8-locus haplotypes (HLA-A-C-B-DRB1-DQA1-DQB1-DPA1-DPB1) by descent. When a parent’s genotype is missing, data of at least two non-HLA identical children were required for the family to be included in the study.

### HLA nomenclature

This report follows the latest HLA nomenclature system for reporting and naming HLA alleles and haplotypes ^36^. The asterisk "*" denotes molecular typing. The digits before the first colon (field 1) indicate the allele group or type. The subtype is indicated by the next set of digits (field 2), while synonymous variants are indicated by the third set of digits (field 3).

### Population genetic analysis

The samples were genotyped at up to the 4th field of resolution. However, statistical population genetic analysis was limited to the 2nd field of resolution to allow for comparisons with previously published reports in other populations. Allele frequencies (A.F.), the degree of heterozygosity, and Guo and Thompson Hardy Weinberg equilibrium (HWE) at a locus-by-locus level were computed using Python for Population Genomics (PyPop v.0.7.0)^37^. The genetic diversity at the allelic level for the UAE cohort was calculated using polymorphism information content (PIC) and power of discrimination (PD) implemented in the FORSTAT tool^38^.

Slatkin’s implementation of the Ewens-Watterson (EW) homozygosity test of neutrality, implemented in PyPop, was performed to examine the effect of natural selection on HLA loci. The test calculated the normalized deviation of homozygosity (Fnd) which is defined as the difference between observed and expected homozygosity divided by the square root of the expected homozygosity’s variance. Haplotypes HLA- A-C-B-DRB1-DQA1-DQB1, HLA-C-B, HLA-DRB1-DQA1-DQB1 and HLA-DPA1-DPB1 were observed and manually counted by the co-authors using MS Excel.

### MHC conserved extended haplotypes (CEHs)

Putative CEHs (extending from HLA-C to HLA-DQB1) were identified through a previously described and established approach^3,8,13,15,19^. A haplotype frequency cut-off of 0.005 is usually used to distinguish a common CEH in a certain population, considering the high level of polymorphism within the MHC^8^. Nonetheless, due to the sample size, a cutoff of 0.02 is used in this study to distinguish CEHs in the current cohort. First, the complete dataset of 170 phased extended 8-locus HLA haplotypes (HLA- A-C-B-DRB1-DQA1-DQB1-DPA1-DPB1) obtained from the segregation analysis were sorted based on HLA-B, HLA-DRB1, and HLA-DQB1 loci respectively using Microsoft Excel. Next, 5-locus haplotypes (HLA- C-B-DRB1-DQA1-DQB1) Haplotypes that were observed at least 5 times were extracted for further analysis of CEH. Novel CEH were named according to a previously described system by Degli-Esposti, et al. ^19^, in which the CEH is identified by its HLA-B allele type, followed by a sequential number indicating its order of discovery (e.g., 18.1, 18.2, 18.3).

### Analysis of genetic relationships with other populations

A Principal Component Analysis (PCA) plot and a phylogenetic tree were generated for 50 populations including the cohort studies herein, with high-resolution genotypes of HLA-A, HLA-B, and HLA-DRB1. Those loci were chosen as they exhibit the greatest level of heterogeneity, effectively representing world populations while simultaneously expanding the number of datasets available for the analysis. The world populations datasets were obtained from the Allele Frequency Net Database (AFND)^39^. The populations were selected from different world regions including the Middle East, Central and South Asia, Sub-Saharan Africa, North Africa, Oceania, South America, East Asia, and Europe. The world populations datasets were chosen only if they satisfy the gold and silver quality standard based on AFND criteria^39^. The PCA was conducted using IBM SPSS Statistics 19 software (IBM Corporation, Armonk, NY, USA). The phylogenetic tree was constructed using the neighbour-joining (NJ) clustering method implemented in POPTREEW. The distance was set to Nei's genetic distance (DA), and the Bootstrap to 1,000 replications.

## Results

### HLA allele and MHC haplotype frequencies: genetic similarity with other populations

The current cohort included 40 two-generation and one three-generation families from the UAE (see Table S1). In total, 170 phased HLA- A-C-B-DRB1-DQA1-DQB1-DPA1-DPB1 haplotypes were described by segregation analysis. Ten haplotypes were obtained from the three-generation family (referred to as HF8); 4 from the grandparents, and 6 from 3 individuals who married into the family. Ambiguities and allelic dropout in parental genotypes were resolved by inference from offspring. Only one and three genotypes were missing from HLA-DQA1 and HLA-DQB1 respectively, due to sequencing error.

HLA class I and class II allele count, and frequencies are listed in Tables 1 and 2. Cumulatively, 31 different alleles were observed in HLA-A, 29 in HLA-C, 41 in HLA-B, 30 in HLA-DRB1, 13 in HLA-DQA1, and 15 in HLA-DQB1. The most frequent alleles were HLA-A*02:01 (A.F. 0.15), HLA-C*04:01 (A.F. 0.19), HLA-B*51:01 (A.F. 0.12), HLA-DRB1*03:01 (A.F. 0.29), HLA-DQA1*05:01 (A.F. 0.28), HLA-DQB1*02:01 (A.F. 0.29), HLA-DPA1*01:03 (A.F. 0.67), and HLA-DPB1*04:01 (A.F. 0.31).

**Table 1:** HLA class I (HLA-A, HLA-C, HLA-B) allelic count and frequencies observed in the UAE cohort. A.F.: allele frequency.

*HLA-A*	*Count*	*A.F.*	*HLA-C*	*Count*	*A.F.*	*HLA-B*	*Count*	*A.F.*
02:01	25	0.15	04:01	32	0.19	51:01	20	0.12
11:01	18	0.11	07:02	21	0.12	08:01	18	0.11
24:02	14	0.08	06:02	20	0.12	40:06	13	0.08
32:01	13	0.08	15:02	18	0.11	50:01	11	0.06
03:01	12	0.07	07:01	10	0.06	18:01	10	0.06
68:01	12	0.07	16:02	10	0.06	58:01	9	0.05
01:01	11	0.06	03:02	9	0.05	35:01	8	0.05
30:02	10	0.06	12:03	6	0.04	35:03	8	0.05
26:01	9	0.05	08:02	5	0.03	53:01	6	0.04
30:01	7	0.04	12:02	5	0.03	35:08	5	0.03
33:03	7	0.04	17:01	5	0.03	52:01	5	0.03
23:01	6	0.04	15:05	4	0.02	07:02	4	0.02
03:02	4	0.02	01:02	2	0.01	14:02	4	0.02
29:02	3	0.02	02:02	2	0.01	41:01	4	0.02
31:01	2	0.01	03:04	2	0.01	42:01	4	0.02
68:02	2	0.01	07:04	2	0.01	57:01	4	0.02
01:03	1	0.01	14:02	2	0.01	38:01	3	0.02
02:02	1	0.01	15:04	2	0.01	45:01	3	0.02
02:03	1	0.01	15:13	2	0.01	07:05	2	0.01
02:09	1	0.01	16:01	2	0.01	13:01	2	0.01
02:11	1	0.01	02:10	1	0.01	15:10	2	0.01
26:17	1	0.01	02:16	1	0.01	27:03	2	0.01
29:01	1	0.01	03:03	1	0.01	35:02	2	0.01
29:10	1	0.01	04:03	1	0.01	39:01	2	0.01
30:04	1	0.01	07:18	1	0.01	44:02	2	0.01
33:01	1	0.01	08:01	1	0.01	58:02	2	0.01
34:02	1	0.01	12:19	1	0.01	13:02	1	0.01
36:01	1	0.01	16:04	1	0.01	14:01	1	0.01
66:02	1	0.01	18:01	1	0.01	15:02	1	0.01
69:01	1	0.01				15:03	1	0.01
74:01	1	0.01				15:17	1	0.01
						15:22	1	0.01
						15:67	1	0.01
						37:01	1	0.01
						40:16	1	0.01
						44:03	1	0.01
						47:03	1	0.01
						51:08	1	0.01
						55:01	1	0.01
						73:01	1	0.01
						81:01	1	0.01

**Table 2:** HLA class II (HLA-DRB1, HLA-DQA1, HLA-DQB1, HLA-DPA1, HLA-DPB1) allelic count and frequencies observed in the UAE cohort. A.F.: allele frequency.

*HLA-DRB1*	*Count*	*A.F.*	*HLA-DQA1*	*Count*	*A.F.*	*HLA-DQB1*	*Count*	*A.F.*	*HLA-DPA1*	*Count*	*A.F.*	*HLA-DPB1*	*Count*	*A.F.*
03:01	49	0.29	05:01	48	0.28	02:01	49	0.29	01:03	114	0.67	04:01	52	0.31
16:02	16	0.09	01:02	42	0.25	05:02	35	0.21	02:01	42	0.25	02:01	34	0.20
16:01	15	0.09	05:05	16	0.09	03:02	18	0.11	02:02	6	0.04	14:01	17	0.10
11:01	12	0.07	03:03	12	0.07	05:01	14	0.08	02:07	3	0.02	04:02	14	0.08
04:05	9	0.05	01:01	11	0.07	03:01	13	0.08	03:01	2	0.01	01:01	7	0.04
07:01	9	0.05	03:01	11	0.07	02:02	10	0.06	01:04	1	0.01	03:01	7	0.04
01:01	6	0.04	01:03	9	0.05	06:01	8	0.05	01:14	1	0.01	13:01	6	0.04
04:02	6	0.04	02:01	7	0.04	04:02	6	0.04	02:09	1	0.01	17:01	6	0.04
01:02	6	0.03	01:05	4	0.02	06:02	6	0.04				104:01	6	0.04
11:04	5	0.03	01:04	3	0.02	05:03	3	0.02				10:01	4	0.02
15:01	5	0.03	04:01	3	0.02	03:19	1	0.01				18:01	4	0.02
15:02	4	0.02	03:02	2	0.01	03:27	1	0.01				09:01	3	0.02
15:03	4	0.02	05:09	1	0.01	03:35	1	0.01				105:01	2	0.01
10:01	3	0.02				06:03	1	0.01				107:01	2	0.01
03:02	2	0.01				06:04	1	0.01				15:01	1	0.01
04:03	2	0.01										26:01	1	0.01
04:06	2	0.01										39:01	1	0.01
09:01	2	0.01										45:01	1	0.01
13:01	2	0.01										91:01	1	0.01
03:07	1	0.01										124:01	1	0.01
04:01	1	0.01												
04:04	1	0.01												
08:04	1	0.01												
11:02	1	0.01												
13:02	1	0.01												
13:03	1	0.01												
14:04	1	0.01												
14:15	1	0.01												
14:21	1	0.01												
15:06	1	0.01												

Overall, no deviation from HWE was observed except for HLA-DQB1 (Table S2). The PIC and PD for HLA-A, HLA-C, HLA-B, HLA-DRB1, HLA-DQA1, and HLA-DQB1 were calculated to measure the extent of genetic diversity within the cohort (Table S2). The HLA class I loci were relatively more diverse compared to the HLA class II loci with HLA-B being the most polymorphic locus at a PIC of 0.94 and HLA-DQA1 being the least polymorphic locus with a PIC of 0.82. A PD value greater than 0.80 is indicative of a high degree of polymorphism^40^. The results of the EW homozygosity test of neutrality are summarized in Table S3. A large negative Fnd value suggests that the observed homozygosity is skewed toward balancing selection, while a strong positive value implies directional selection. From the results, only the HLA-DRB1 locus showed a slight directional selection. The two loci HLA-DPA1 and HLA-DPB1 were excluded from the HWE, PIC, PD, and EW homozygosity analyses.

From HLA class I, the most frequent HLA-C-B two-locus haplotype was HLA- C*07:02-B*08:01 (H.F. 0.094) (Table 3). From HLA class II, HLA-DRB1*03:01-DQA1*05:01-DQB1*02:01 (H.F. 0.253), and HLA-DPA1*01:03-DPB1*04:01 (H.F. 0.276) were the most frequent HLA-DRB1-DQA1-DQB1 (Table 4) and HLA-DPA1-DPB1 haplotypes (Table 5), respectively. Please refer to supplementary Tables S4–S6 for the complete list of the HLA-C-B, HLA-DRB1-DQA1-DQB1 and HLA-DPA1-DPB1 frequencies.

**Table 3:** Five most frequent HLA-C-B two-locus haplotype counts observed in the UAE cohort. H.F.: haplotypes frequency.

Haplotypes				Count (n = 170)	H.F.
HLA- C*	*07:02*	-B*	*08:01*	16	0.094
HLA- C*	*15:02*	-B*	*40:06*	12	0.071
HLA- C*	*06:02*	-B*	*50:01*	10	0.059
HLA- C*	*03:02*	-B*	*58:01*	8	0.047
HLA- C*	*04:01*	-B*	*35:03*	8	0.047

**Table 4:** Five most frequent HLA-DRB1-DQA1-DQB1 three-locus haplotype counts observed in the UAE cohort. H.F.: haplotypes frequency.

Haplotype						Count (N = 170)	H.F.
HLA- DRB1*	*03:01*	-DQA1*	*05:01*	-DQB1*	*02:01*	43	0.253
HLA- DRB1*	*16:01*	-DQA1*	*01:02*	-DQB1*	*05:02*	15	0.088
HLA- DRB1*	*16:02*	-DQA1*	*01:02*	-DQB1*	*05:02*	14	0.082
HLA- DRB1*	*11:02*	-DQA1*	*05:05*	-DQB1*	*03:01*	7	0.041
HLA- DRB1*	*04:05*	-DQA1*	*03:03*	-DQB1*	*03:02*	6	0.035

**Table 5:** Six most frequent HLA-DPA1-DPB1 two-locus haplotype counts observed in the UAE cohort. H.F.: haplotypes frequency.

Haplotype				Count (N = 170)	H.F.
HLA- DPA1*	*01:03*	-DPB1*	*04:01*	47	0.276
HLA- DPA1*	*01:03*	-DPB1*	*02:01*	32	0.188
HLA- DPA1*	*02:01*	-DPB1*	*14:01*	15	0.088
HLA- DPA1*	*01:03*	-DPB1*	*04:02*	14	0.082
HLA- DPA1*	*01:03*	-DPB1*	*104:01*	6	0.035
HLA- DPA1*	*02:01*	-DPB1*	*17:01*	6	0.035

**Figure 1:**
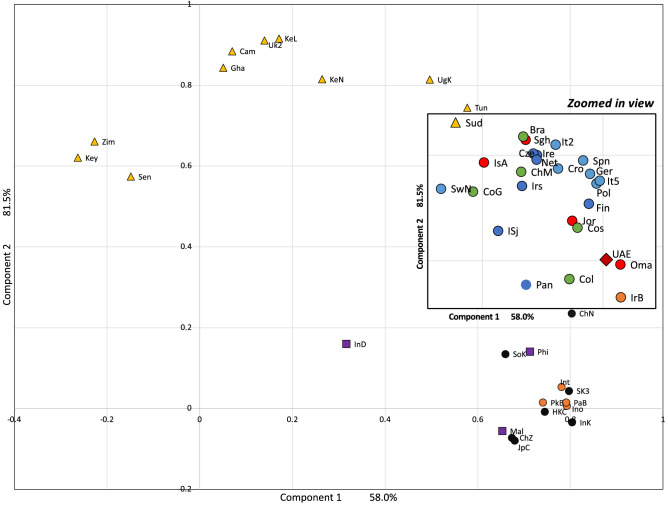
Principal Component Analysis (PCA) for 50 populations (including the UAE cohort reported herein) from different world regions calculated using HLA-A, -B, and -DRB1 loci. The first component is explained by 58.0% of the variance, while the second component is described by 81.5% of the total variance. The Sub-Sharan Africa populations are denoted in yellow triangles, while European populations are represented by light blue dots; the Middle Easter populations are presented in red dots; the Oceania populations are in purple squares; the South Asian populations are indicated by orange dots; black dots were assigned to East Asian populations and green dots to South American groups. For the complete PCA plot and description of datasets used and their abbreviations, refer to Table S7.

**Figure 2:**
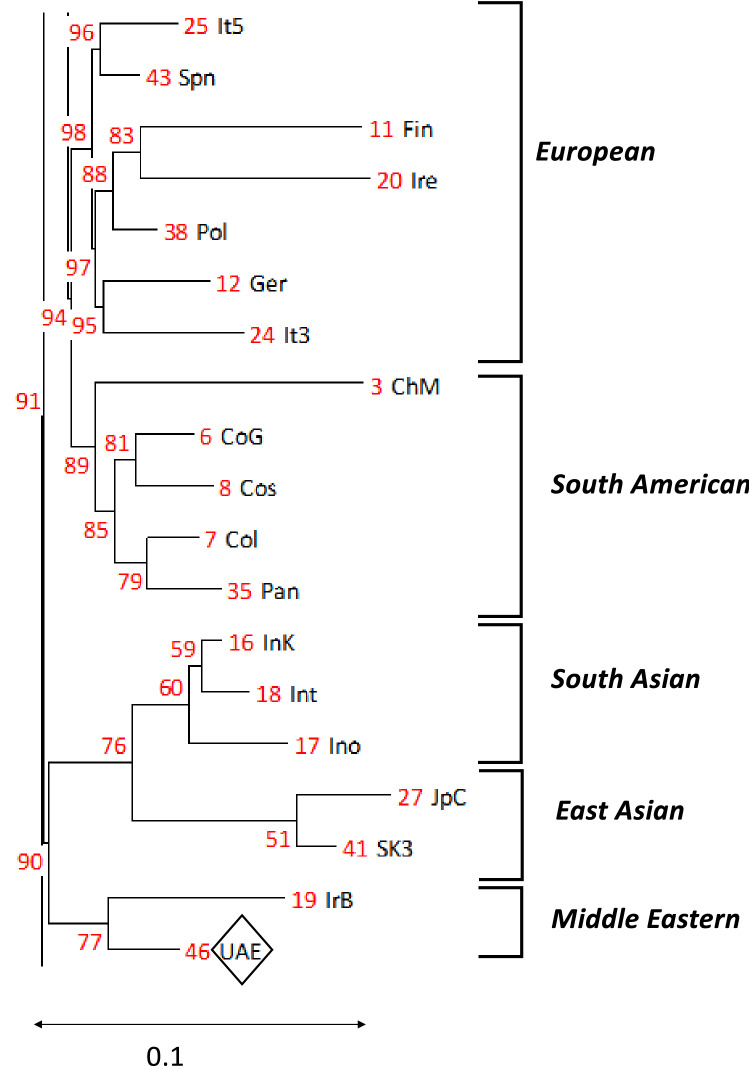
A zoom in view of the neighbor-joining phylogenetic tree showing relatedness between the UAE population and other populations calculated using HLA-A, -B and -DRB1 loci. For the complete phylogenetic tree and description of datasets used and their abbreviations, refer to Table S7.

The PCA plot shown in Fig. 1 shows that the UAE clusters with the Omani population (abbreviated as ‘Oma’) and the Baloch subpopulation of Iran (abbreviated as ‘IrB’), and then South American and European populations (with some proximity to East Asian populations). Similarly, the phylogenetic tree in Fig. 2 shows that the UAE population is genetically close to the Baloch subpopulation of Iran. Description and reference for each population dataset used in the PCA and phylogenetic tree are listed in Table S7.

### Identification of HLA conserved extended haplotypes

The complete list of the phase-segregated 5-locus MHC haplotypes (HLA-C-B-DRB1-DQA1-DQB1) observed in the current UAE cohort is presented in Table S8. To allow for a more rigorous identification of MHC CEHs in the UAE population, only CEHs with H.F. > 0.02, are described and discussed hereafter (See Table 6). Those include HLA- C*07:02-B*08:01-DRB1*03:01-DQA1*05:01-DQB1*02:01 (H.F. 0.094), HLA- C*15:02-B*40:06-DRB1*16:02-DQA1*01:02-DQB1*05:02 (H.F. 0.035), HLA- C*16:02-B*51:01-DRB1*16:01-DQA1*01:02-DQB1*05:02 (H.F. 0.029), HLA- C*03:02-B*58:01-DRB1*03:01-DQA1*05:01-DQB1*02:01 (H.F. 0.024), and HLA- C*03:02-B*58:01-DRB1*16:01-DQA1*01:02-DQB1*05:02 (H.F. 0.024).

**Table 6:** MHC conserved extended haplotypes (CEH) derived from 41 UAE families with H.F. > 0.02. H.F.: haplotypes frequency.

Conserved Extended Haplotypes (CEH)									n (N = 170)	H.F.
HLA- C*07:02	–	B*08:01	–	DRB1*03:01	–	DQA1*05:01	–	DQB1*02:01	16	0.094
HLA- C*15:02	–	B*40:06	–	DRB1*16:02	–	DQA1*01:02	–	DQB1*05:02	6	0.035
HLA- C*16:02	–	B*51:01	–	DRB1*16:01	–	DQA1*01:02	–	DQB1*05:02	5	0.029
HLA- C*03:02	–	B*58:01	–	DRB1*03:01	–	DQA1*05:01	–	DQB1*02:01	4	0.024
HLA- C*03:02	–	B*58:01	–	DRB1*16:01	–	DQA1*01:02	–	DQB1*05:02	4	0.024

When combined, these five CEHs represent 20.6% (35 out of 170) of the haplotype pool in the current UAE cohort. Subsequently, these CEH were analyzed to infer their most probable ancestry (MPA) based on previously published frequencies in African, Asian, and Caucasian populations^41^. MPA is based on evaluating the existence of distinctive ethnic/region-specific CEH in the relevant continental such that CEHs that are generally present in high frequency (e.g., H.F. > 0.10) in a particular non-recently admixed human continental group were regarded to be indicative of that regional origin. Table S9 provides the names for the CEHs observed in the study.

HLA-B*08:01 (A.F. 0.11) was the most common allele inherited as part of the haplotype block HLA- C*07:02-B*08:01-DRB1*03:01-DQA1*05:01-DQB1*02:01 (H.F. 0.094) (Table 7). The most common HLA-A alleles linked to the HLA- C*07:02-B*08:01-DRB1*03:01-DQB1*02:01 CEH in the UAE cohort were HLA-A*26:01 (31.3%), HLA-A*68:01 (25.0%), HLA-A*24:02 (18.8%), *HLA-68:01* (6.3%), HLA-A*02:01 (6.3%), HLA-A*03:02 (6.3%) and HLA-A*11:01 (6.3%), See Table 7. This CEH was frequently associated with HLA- DPA1*01:03-DPB1*02:01 (18.8%) and HLA- DPA1*01:03-DPB1*04:02 (25.0%) haplotype blocks.

**Table 7:** MHC haplotypes of the UAE families selected on the basis of HLA-B*08:01. The group of alleles that make up the designated CEH in column “CEH” are denoted in bold. CEH: conserved extended haplotypes; MPA: Most probable Ancestry; SA: South Asian; C: Caucasian.

Family	Haplotype	*HLA-A*	*HLA-C*	*HLA-B*	*HLA-DRB1*	*HLA-DQA1*	*HLA-DQB1*	*HLA-DPA1*	*HLA-DPB1*	CEH	MPA
DF24	a	26:01:01:01	**07:02:01:01**	**08:01:01**	**03:01:01**	**05:01:01:03**	**02:01:01**	02:01:01	04:01:01	8.2	SA^46,47,54^
DF22	b	26:01:01:01	**07:02:01:01**	**08:01:01**	**03:01:01**	**05:01:01:03**	**02:01:01**	02:01:01	17:01	8.2	SA^46,47,54^
DF13	a	26:01:01:01	**07:02:01:01**	**08:01:01:01**	**03:01:01:03**	**05:01:01:03**	**02:01:01:01**	01:03:01:01	02:01:02:01	8.2	SA^46,47,54^
HF20	b	26:01:01:01	**07:02:01:01**	**08:01:01:01**	**03:01:01:03**	**05:01:01:03**	**02:01:01:01**	02:01:01:02	14:01:01:01	8.2	SA^46,47,54^
DF31	a	26:01:01:01	**07:02:01:01**	**08:01:01:01**	**03:01:01:03**	**05:01:01:03**	**02:01:01**	01:03:01:03	03:01:01:01	8.2	SA^46,47,54^
DF24	d	68:01:01:02	**07:02:01:01**	**08:01:01**	**03:01:01**	**05:01:01:03**	**02:01:01**	01:03:01:04	04:01:01:01	8.2	SA^46,47,54^
HF19	a	68:01:01:02	**07:02:01:01**	**08:01:01:01**	**03:01:01:01**	**05:01:01:03**	**02:01:01:01**	01:03:01:01	02:01:02:05	8.2	SA^46,47,54^
HF7	b	68:01:01:02	**07:02:01:01**	**08:01:01:01**	**03:01:01:03**	**05:01:01:03**	**02:01:01**	01:03:01:04	04:01:01:06	8.2	SA^46,47,54^
HF19	a	68:01:01:02	**07:02:01:01**	**08:01:01:01**	**03:01:01:01**	**05:01:01:03**	**02:01:01:01**	01:03:01:01	02:01:02:05	8.2	SA^46,47,54^
DF23	d	68:02:01:01	**07:02:01:01**	**08:01:01**	**03:01:01**	**05:01:01:03**	**02:01:01**	01:03:01:05	04:02:01:02	8.2	SA^46,47,54^
HF9	a	24:02:01:04	**07:02:01:01**	**08:01:01:01**	**03:01:01:03**	**05:01:01:03**	**02:01:01**	01:03:01:05	04:02:01:02	8.2	SA^46,47,54^
HF9	b	24:02:01:04	**07:02:01:01**	**08:01:01:01**	**03:01:01:03**	**05:01:01:03**	**02:01:01**	01:03:01:05	04:02:01:02	8.2	SA^46,47,54^
HF9	c	24:02:01:05	**07:02:01:01**	**08:01:01:01**	**03:01:01:03**	**05:01:01:03**	**02:01:01**	02:07:01:01	04:01:01:01	8.2	SA^46,47,54^
HF28	c	03:02:01:01	**07:02:01:01**	**08:01:01:01**	**03:01:01:01**	**05:01:01:03**	**02:01:01:01**	01:03:01:03	03:01:01:01	8.2	SA^46,47,54^
HF7	d	02:01:01:01	**07:02:01:01**	**08:01:01:01**	**03:01:01:03**	**05:01:01:03**	**02:01:01**	02:01:01:06	107:01	8.2	SA^46,47,54^
HF29	a	11:01:01:01	**07:02:01:01**	**08:01:01:01**	**03:01:01:03**	**05:01:01:03**	**02:01:01:02**	01:03:01:01	04:02:01:01	8.2	SA^46,47,54^
HF11	d	29:02:01:01	**07:01:01:01**	**08:01:01:01**	**03:01:01:01**	**05:01:01:02**	**02:01:01:01**	02:01:01:02	14:01:01:01	8.1	C^8,15,19^
HF18	b	30:02:01:03	07:01:01:01	08:01:01:01	03:01:01:02	01:03:01:01	06:01:01:01	01:03:01:02	04:01:01:04		

Allele HLA-B*40:06 (A.F. 0.08) was frequently inherited as part of the HLA- C*15:02-B*40:06-DRB1*16:02-DQA1*01:02-DQB1*05:02 CEH (H.F. 0.035). Eighty-Three per cent (83.3%) of this CEH extended to include HLA-A*11:01 (See Table 8). This CEH was associated with HLA- DPA1*01:03-DPB1*02:01 (33.3%), HLA- DPA1*01:03-DPB1*04:02 (16.7%), HLA- DPA1*01:03-DPB1*04:02 (16.7%), HLA- DPA1*01:03-DPB1*18:01 (16.7%), HLA- DPA1*01:03-DPB1*04:01 (16.7%), and HLA- DPA1*02:01-DPB1*14:01 (16.7%).

**Table 8:** MHC haplotypes of UAE families marked by HLA-B*40:06. The group of alleles that make up the designated CEH in column “CEH” are denoted in bold. ^‡^Proposed.

Family	Haplotype	*HLA-A*	*HLA-C*	*HLA-B*	*HLA-DRB1*	*HLA-DQA1*	*HLA-DQB1*	***HLA-DPA1***	***HLA-DPB1***	**CEH**
HF6	a	11:01:01:01	15:02:01:01	40:06:01:02	03:01:01:03	05:01:01:03	02:01:01:01	01:03:01:04	04:01:01:04	
HF38	d	11:01:01:01	15:02:01:01	40:06:01:02	10:01:01:03	01:05:01:01	05:01:01:05	01:03:01:01	02:01:02:28	
HF16	b	11:01:01:01	15:02:01:01	40:06:04:01	14:04:01:02	01:04:01:02	05:03:01:01	01:03:01:01	02:01:02:01	
HF30	b	11:01:01:01	15:02:01:01	40:06:01:02	16:01:01	01:02:02:01	05:02:01:01	02:01:01:02	14:01:01:01	
HF36	b	01:01:01:01	**15:02:01:01**	**40:06:01:02**	**16:02:01:02**	**01:02:02:01**	**05:02:01**	01:03:01:02	18:01:01:01	60.4^‡^
DF2	d	11:01:01:01	**15:02:01:01**	**40:06:01:02**	**16:02:01:02**	**01:02:02**	**05:02:01**	01:03:01:04	04:01:01	60.4^‡^
HF24	a	11:01:01:01	**15:02:01:01**	**40:06:01:02**	**16:02:01:02**	**01:02:02:01**	**05:02:01:01**	01:03:01:01	02:01:02:01	60.4^‡^
HF8	i	11:01:01:01	**15:02:01:01**	**40:06:01:02**	**16:02:01:02**	**01:02:02:01**	**05:02:01:01**	01:03:01:01	04:02:01:02	60.4^‡^
HF29	b	11:01:01:01	**15:02:01:01**	**40:06:01:02**	**16:02:01:02**	**01:02:02:01**	**05:02:01:01**	01:03:01:05	02:01:02:03	60.4^‡^
HF25	d	11:01:01:01	**15:02:01:01**	**40:06:01:02**	**16:02:01:02**	**01:02:02:01**	**05:02:01:01**	02:01:01:02	14:01:01:01	60.4^‡^

HLA-B*51:01 (A.F. 0.12) allele was the most frequent HLA-B allele in the current cohort, and it was frequently observed as part of the HLA- C*16:02-B*51:01-DRB1*16:01-DQA1*01:02-DQB1*05:02 CEH (H.F. 0.029) (See Table 9). This CEH was either associated with HLA-A*32:01 (60%) or HLA-A*02:01 (40%) and extended to include HLA-DPA1*01:03-DPB1*02:01.

**Table 9:** MHC haplotypes of UAE families selected on the basis of HLA-B*51:01. The group of alleles that make up the designated CEH in column “CEH” are denoted in bold. ^‡^Proposed*.*

Family	Haplotype	*HLA-A*	*HLA-C*	*HLA-B*	*HLA-DRB1*	*HLA-DQA1*	*HLA-DQB1*	*HLA-DPA1*	*HLA-DPB1*	CEH
HF39	b	02:01:01:01	**16:02:01:01**	**51:01:01:01**	**16:01:01**	**01:02:02:01**	**05:02:01:01**	01:03:01:01	02:01:02:05	51.2^‡^
HF22	a	02:01:01:01	**16:02:01:01**	**51:01:01:01**	**16:01:01**	**01:02:02:01**	**05:02:01:01**	01:03:01:01	02:01:02:05	51.2^‡^
HF8	a	32:01:01:01	**16:02:01:01**	**51:01:01:01**	**16:01:01**	**01:02:02:01**	**05:02:01:01**	01:03:01:01	02:01:02:01	51.2^‡^
HF8	f	32:01:01:01	**16:02:01:01**	**51:01:01:01**	**16:01:01**	**01:02:02:01**	**05:02:01:01**	01:03:01:01	02:01:02:01	51.2^‡^
HF8	j	32:01:01:01	**16:02:01:01**	**51:01:01:01**	**16:01:01**	**01:02:02:01**	**05:02:01:01**	01:03:01:05	02:01:02:01	51.2^‡^

The HLA-B*58:01 allele (A.F. 0.05) was associated with two different CEHs including the East Asian CEH 58.1 (HLA- C*03:01-B*58:01-DRB1*03:01-DQA1*05:01-DQB1*02:01)^15^, and HLA- C*03:02-B*58:01-DRB1*16:01-DQA1*01:02-DQB1*05:02 (Table 10). Both haplotypes were associated with the same class I haplotype block (HLA- A*33:03-C*03:02-B*58:01). Fifty per cent of the 58.1 CEHs were associated with HLA- DPA1*02:02-DPB1*13:01, while HLA- DPA1*01:03-DPB1*04:01 haplotype was associated with 50% of HLA- C*03:02-B*58:01-DRB1*16:01-DQA1*01:02-DQB1*05:02 CEH observed.

**Table 10:** MHC haplotypes of UAE families marked by HLA-B*58:01. The group of alleles that make up the designated CEH in column “CEH” are denoted in bold. CEH: conserved extended haplotypes; MPA: Most probable Ancestry; EA: East Asia; ^‡^Proposed*.*

Family	Haplotype	*HLA-A*	*HLA-C*	*HLA-B*	*HLA-DRB1*	*HLA-DQA1*	*HLA-DQB1*	*HLA-DPA1*	*HLA-DPB1*	CEH	MPA
HF26	c	02:01:01:01	**03:02:02:01**	**58:01:01:01**	**03:01:01:01**	**05:01:01:03**	**02:01:01:01**	02:01:01:02	14:01:01:01	58.1	EA^5^
HF10	d	23:01:01:01	**03:02:02:01**	**58:01:01:01**	**03:01:01:01**	**05:01:01:03**	**02:01:01:01**	02:01:01:03	17:01:01:01	58.1	EA^5^
HF17	b	33:03:01:01	**03:02:02:01**	**58:01:01:01**	**03:01:01:01**	**05:01:01:03**	**02:01:01:01**	01:03:01:02	04:01:01:04	58.1	EA^5^
HF7	a	33:03:01:01	**03:02:02:01**	**58:01:01:01**	**03:01:01:03**	**05:01:01:03**	**02:01:01**	01:03:01:02	04:01:01:01	58.1	EA^5^
DF14	d	33:03:01:01	**03:02:02:01**	**58:01:01:01**	**16:01:01**	**01:02:02**	**05:02:01**	02:02:02	13:01:01	58.2^‡^	
HF39	d	33:03:01:01	**03:02:02:01**	**58:01:01:01**	**16:01:01**	**01:02:02:01**	**05:02:01:01**	01:03:01:04	04:01:01:04	58.2^‡^	
HF1	a	33:03:01:01	**03:02:02:01**	**58:01:01:01**	**16:01:01**	**01:02:02:01**	**05:02:01:01**	02:01:01:02	14:01:01:01	58.2^‡^	
DF14	d	33:03:01	**03:02:02:01**	**58:01:01:01**	**16:01:01**	**01:02:02**	**05:02:01**	02:02:02	13:01:01	58.2^‡^	

## Discussion

The first whole genomes analysis of two UAE nationals^42,43^ has provided insights into the genomic structure and the putative genetic origins of its population. Following that, a comprehensive, large-scale stratification study of the UAE population concluded that genetic admixture throughout the Arabian Peninsula's eastern shore and south-eastern tip happened gradually and without clear social stratification boundaries^43^. This, and another mitogenome study^44^, have shown that there was no apparent association between birthplace and ancestral background, indicating that the contemporary UAE population developed over generations prior to the establishment of the current political borders with a significant genetic influence from the Middle East, Central/South Asia, and Sub-Sahara^43^.

Conserved extended haplotypes (CEHs) of the MHC, and their fragments, have been shown to be useful as markers for disease association, immune response, and anthropology. This study describes the diversity of MHC CEHs derived from 41 UAE families. As in the previously cited publications, the data presented herein suggest evidence of gene flow from neighbouring ethnic groups in the contemporary UAE population.

Overall, the most prevalent HLA class I allele lineages reported [e.g., HLA-A*02 (A.F. 15.30%), HLA-A*11 (A.F. 10.60%), HLA-C*04 (A.F. 19.40%), HLA-C*06 (A.F. 11.80%), HLA-C*07 (A.F. 20.10%), HLA-B*08 (A.F. 10.60%), HLA-B*50 (A.F. 6.50%) and HLA-B*51 (A.F. 11.80%)] are consistent with previous reports on the UAE population using PCR-SSP methods^45^.

The current study detected 5 putative CEHs in the current UAE population, three of which were identified as novel CEHs. Overall, the aggregate percentage of those 5 putative CEHs was 20.6%.

As noted earlier, HLA-B is the most polymorphic HLA locus. Thus, individual CEHs will be discussed hereafter based on the relevance of the HLA-B allele each CEH contains.

The examination of the MHC CEHs in the current cohort has revealed that HLA-B*08:01 (A.F. 0.11), the second most frequent HLA-B allele, commonly marked the HLA- C*07:02-B*08:01-DRB1*03:01-DQA1*05:01-DQB1*02:01 CEH, which extended to include HLA-A*26:01 (31.3%), *HLA-68:01* (25.0%), and HLA-A*24:02 (18.8%). This CEH, previously assigned as 8.2 by Witt, et al. ^46^ in Northern Indians, differs from the Caucasian 8.1 at the HLA-C locus, in the complement region, and by several repeat units at most microsatellite loci. Hence, it has been suggested that the two CEHs are not derived from one another^46,47^. This CEH was also found to be commonly associated with HLA-A*26:01in Asian Indians^47^. The 8.2 CEH was also observed at 2.68% in Kuwaiti unrelated subjects^13^, and 3.00% in unrelated Saudi Arabian bone marrow donors^32^.

Of the total number of HLA- C*07:02-B*08:01-DRB1*03:01-DQA1*05:01-DQB1*02:01 CEHs observed, 25% were extended to HLA-A*68:01. The association of the 8.2 CEH with the HLA-A*68:01 allele has not been identified in South Indians. Nonetheless, the HLA-A*68:01 allele has been found to be highly prevalent in Native Americans^48^ and Africans^49^, whereas it is found to be at low levels in Southeast Asia^50^. A genome-wide study of populations of the Arabian Peninsula demonstrated a Sub-Saharan African input of only 4.0% by 1,754 Common Era (CE) in a cohort from the UAE^51^. Therefore, it can be argued that HLA-A*68:01 was introduced to the UAE from a Sub-Saharan founder, considering that both West and East African populations were transported to the Middle East, Arabia, and the Indian Ocean during the 15th to 19th centuries during a time when the slave trade was common^52^. HLA-A*68:01 is of particular interest due to several unusual features, such as its weak binding affinity to CD8 and its ability to bind unusual long peptides because of peptide bending in the binding groove^53^.

Overall, 88.9% of the HLA-B*08:01 alleles observed were part of CEHs identified in South Asians^46,47,54^. On the other hand, however, one family (Family IDs: HF11) carried the Caucasian 8.1 CEH, implying a possible Caucasian origin (Table 7).

According to the IMGT/HLA database HLA-B*40 is one of the most polymorphic lineages of HLA antigens^55^. However, only two HLA-B*40 subtypes were identified in this study, specifically HLA-B*40:06 (7.60%) and HLA-B*40:16 (0.60%). The second most prevalent haplotype in the current cohort CEH HLA- C*15:02-B*40:06-DRB1*16:02-DQA1*01:02-DQB1*05:02 extended to include HLA-A*11:01. Interestingly, unlike the other CEH in this study, class I fragments of this haplotype (HLA- A*11:01-C*15:02-B*40:06) were also observed (Table 8). CEHs were initially described using serological methods, where the HLA-B*40:01 allele is recognized by B60 antigen serotype^13^. Subsequently, CEHs that included HLA-B*40:01 such as HLA- C*03:04-B*40:01-DRB1*04:04-DQA1*03:01-DQB1*03:02, HLA- C*03:04-B*40:01-DRB1*08:01-DQA1*04:01-DQB1*04:02, and HLA- C*03:04-B*40:01-DRB1*13:02-DQA1*01:02-DQB1*06:04 were named 60.1, 60.2, and 60.3, respectively. In this regard, we suggest referring to the current CEH (HLA- C*15:02-B*40:06-DRB1*16:02-DQA1*01:02-DQB1*05:02) as 60.4. This CEH also existed, at a frequency as low as 0.92% and 1.10%, in a cohort from Kuwait^30^ and the Balouch group in Iran^56^, respectively.

Although HLA-B*51:01 (A.F. 0.118) is the most frequent HLA-B allele in the current cohort, it was only observed in a single CEH, HLA- C*16:02-B*51:01-DRB1*16:01-DQA1*01:02-DQB1*05:02 (unlike HLA-B*08:01 or even HLA-B*58:01). This CEH also extended to include HLA-A*32:01 and HLA-DPA1*01:03:01-DPB1*02:01. We suggest referring to this CEH as 51.2 since CEH 51.1 has been previously reported^15,57^. The HLA-B*51 allele is considered the risk factor for Behçet’s disease, a disease that has a strong geographical prevalence distribution along the ancient Silk Road which ran from the Mediterranean to Northern China^58^. Therefore, the prevalence of Behçet’s is highest among populations of Japan, China, Korea, Turkey, Iran, Tunisia, and other Middle Eastern countries^59^, whereas it is low in Africa, Oceania, and South America, where the frequency of the HLA-B*51 allele is low^60,61^.

HLA-B*58:01 (A.F. 0.05) was associated with two different CEH, the East Asian 58.1 CEH^5,15^ (HLA- C*03:02-B*58:01-DRB1*03:01-DQA1*05:01-DQB1*02:01), and HLA- C*03:02-B*58:01-DRB1*16:01-DQA1*01:02-DQB1*05:02, which both extended to include HLA-A*33:03. We suggest that the latter be referred to as 58.2. Both CEH 58.1 and 58.2 only differed in their HLA- DRB1-DQA1-DQB1 haplotype. The 58.1 CEH was associated with HLA- DRB1*03:01-DQA1*05:02-DQB1*02:01, similar to 8.2 CEH, whereas 58.2 shared the same HLA- DRB1*16:01-DQA1*01:02-DQB1*05:02 with CEH 51.2.

The East Asian 58.1 and its recombinants were also observed at high frequency in people from the Arabian Peninsula ^31,32,39^, as well as South Asia^46^, but not in Caucasians^8^, indicating a possible genetic link with populations from East Asia. This can be supported by historical documents which indicate that bidirectional trade movements from Central and South Asia through the Arabian Gulf into the Arabian peninsula's south-eastern region, which currently includes the UAE, were feasible and did occur^62^. Furthermore, as evident by autosomal Short Tandem Repeats (STRs) genotyping, this cultural diffusion from Arabia has shaped worldwide Muslim populations in Asia including the Thai-Malay^63^ and Chinese Muslim populations^64^. Furthermore, analysis by autosomal STRs^65^, mitochondrial DNA^66^, and Y-chromosomes^67^ have revealed that historically attested movements into the Indian subcontinent have accounted for a cultural diffusion as well as a minor but detectable gene flow from West Asia and Arabia.

Natural selection^3,8,17^ is often considered an important component in the evolution of the MHC and the production of CEHs. However, evident by the information presented here and other reports^42–44^, it seems that the MHC genomic landscape of the contemporary UAE nationals must have also been shaped by both transcontinental migration between Africa, Asia, and Europe, which involved a diverse array of ethnic groupings^34,51^, and the nomadic lifestyles of some Arabian communities, notably the Bedouins.

HLA allele frequency as genetic estimators were shown to have the ability to mimic the results obtained with genome-wide data for PCA^6^. In the current study, high resolution and quality HLA allele frequency data from Middle Eastern populations were scarce, which may have resulted in an imbalance of the clustering pattern in the PCA plot (Fig. 1) and the phylogenetic tree (Fig. 2). The analysis of the genetic relationship between the current UAE dataset with world populations using PCA and the phylogenetic tree seem to provide significantly different qualitative findings from one another. Additionally, the identified CEH and their ethnic identities observed in the current cohort do not seem to correlate with the results of the PCA plot or the phylogenetic tree. We argue that the direction of the gene flow at the CEH level (whether it is from East to West or vice versa) requires additional evaluations of the whole Asian continent from the Arabian Peninsula to north-eastern Siberia, and from the northern Urals to Southeast Asia.

High-resolution HLA typing and haplotyping are critical in hematopoietic stem cell transplantation for both unrelated and related donors, particularly in reducing post-transplantation adverse outcomes^68,69^. It is noted that a single high-resolution HLA mismatch may have the same negative effect on outcomes as a low-resolution one^70,71^. As a result, high-resolution HLA typing to lower the probability of missing a clinically important mismatch has been proposed^68^. To this end, data presented herein provide a framework for donor selection during organ and bone marrow transplantation, as well as the identification of permitted mismatches disease risk markers.

Previously, results generated from this laboratory on UAE families with Type 1 Diabetes identified two CEHs (namely 8.2 and 50.2) that have been previously associated with the disease in a neighbouring Indian population^54^. Likewise, several alleles and CEHs associated with autoimmunity and related conditions in other genetically related populations have been identified with high frequency in the current cohort. In this context, further research could be directed into comparing the influence of established HLA autoimmune diseases associations in Arabs using pedigree-based analysis. For example, all the Indian 8.2 CEHs identified herein were intact and therefore present a good model for recombination and disease association mapping.

Further investigation can be carried out in a larger sample size in addition to genotyping different marker catalogues including non-HLA genes (e.g. MICA, MICB, TNF, C2, Bf, C4, among others), microsatellite markers, and polymorphic Alu insertions (POALINs)^72–74^ across the MHC of the UAE populations to ascertain the degree of similarities to other haplotypes of the same CEH blocks, measure the sizes of DNA blocks that may be fixed, and map the recombination hotspots.

## Conclusion

Despite being based on a limited number of haplotypes, this preliminary report identified conserved extended HLA haplotypes in UAE populations and presented evidence of the presence of shared CEHs between the UAE Arab population and other neighboring populations. To the best of our knowledge, this is the first attempt to identify CEH in Arabs using high-resolution HLA pedigree-phased haplotypes.

## Supplementary Information

Supplementary Tables.

Supplementary Figures.

## Data Availability

Data presented herein have been deposited in the NCBI BioSample database (ncbi.nlm.nih.gov/biosample) under the accession number SAMN24578981 to SAMN24579021.

## References

[1] Trowsdale J, Knight JC (2013). Major histocompatibility complex genomics and human disease. Annu. Rev. Genomics Hum. Genet..

[2] Shiina T, Hosomichi K, Inoko H, Kulski JK (2009). The HLA genomic loci map: Expression, interaction, diversity and disease. J. Hum. Genet..

[3] Dawkins R (1999). Genomics of the major histocompatibility complex: Haplotypes, duplication, retroviruses and disease. Immunol. Rev..

[4] Alper CA, Larsen CE (2017). Pedigree-defined haplotypes and their applications to genetic studies. Methods Mol. Biol..

[5] Cheong KY (2001). Localization of central MHC genes influencing type I diabetes. Hum. Immunol..

[6] Barquera R (2020). Diversity of HLA Class I and Class II blocks and conserved extended haplotypes in Lacandon Mayans. Sci Rep.

[7] Meyer, D., VR, C. A., Bitarello, B. D., DY, C. B. & Nunes, K. A genomic perspective on HLA evolution. *Immunogenetics* **70**, 5–27, doi:10.1007/s00251-017-1017-3 (2018). 10.1007/s00251-017-1017-3 PMC5748415 28687858

[8] Yunis EJ (2003). Inheritable variable sizes of DNA stretches in the human MHC: Conserved extended haplotypes and their fragments or blocks. Tissue Antigens.

[9] Alper CA (1976). Inherited structural polymorphism in human C2: Evidence for genetic linkage between C2 and Bf. J Exp Med.

[10] Alper CA, Awdeh ZL, Raum DD, Yunis EJ (1982). Extended major histocompatibility complex haplotypes in man: Role of alleles analogous to murine t mutants. Clin. Immunol. Immunopathol..

[11] Alper CA, Raum D, Karp S, Awdeh ZL, Yunis EJ (1983). Serum Complement ‘Supergenes' of the Major Histocompatibility Complex in Man (Complotypes). Vox Sang.

[12] Alper CA (2021). The Path to Conserved Extended Haplotypes: Megabase-Length Haplotypes at High Population Frequency. Front Genet.

[13] Degli-Esposti MA (1992). Ancestral haplotypes reveal the role of the central MHC in the immunogenetics of IDDM. Immunogenetics.

[14] Gaudieri S, Leelayuwat C, Tay GK, Townend DC, Dawkins RL (1997). The major histocompatability complex (MHC) contains conserved polymorphic genomic sequences that are shuffled by recombination to form ethnic-specific haplotypes. J Mol Evol.

[15] Dorak MT (2006). Conserved extended haplotypes of the major histocompatibility complex: Further characterization. Genes Immun.

[16] Ketheesan N (1999). Reconstruction of the block matching profiles. Hum. Immunol..

[17] Alper C, Awdeh Z, Yunis E (1992). Conserved, extended MHC haplotypes. J Experimental clinical immunogenetics.

[18] Dawkins RL (1983). Disease Associations with Complotypes, Supratypes and Haplotypes. Immunol. Rev..

[19] Degli-Esposti MA (1992). Ancestral haplotypes: Conserved population MHC haplotypes. Hum. Immunol..

[20] Tay GK (1997). Conservation of ancestral haplotypes telomeric of HLA-A. Eur. J. Immunogenet..

[21] Dawkins RL, Lloyd SS (2019). MHC Genomics and Disease: Looking Back to Go Forward. Cells.

[22] Bertaina A, Andreani M (2018). Major histocompatibility complex and hematopoietic stem cell transplantation: Beyond the classical HLA polymorphism. Int. J. Mol. Sci..

[23] Fleischhauer K (2019). Selection of matched unrelated donors moving forward: From HLA allele counting to functional matching. Hematology.

[24] Sirugo G, Williams SM, Tishkoff SA (2019). The missing diversity in human genetic studies. Cell.

[25] Giani AM, Gallo GR, Gianfranceschi L, Formenti G (2020). Long walk to genomics: History and current approaches to genome sequencing and assembly. Comput. Struct. Biotechnol. J..

[26] Al Naqbi H, Mawart A, Alshamsi J, Al Safar H, Tay GK (2021). Major histocompatibility complex (MHC) associations with diseases in ethnic groups of the Arabian Peninsula. Immunogenetics.

[27] Popejoy AB, Fullerton SM (2016). Genomics is failing on diversity. Nature.

[28] Tay GK, Witt CS, Christiansen FT, Corbett JM, Dawkins RL (1995). The identification of MHC identical siblings without HLA typing. Exp. Hematol..

[29] Hajjej A, Saldhana FL, Dajani R, Almawi WY (2020). HLA-A, -B, -C, -DRB1 and -DQB1 allele and haplotype frequencies and phylogenetic analysis of Bahraini population. Gene.

[30] Ameen R, Al Shemmari SH, Marsh SGE (2020). HLA Haplotype Frequencies and Genetic Profiles of the Kuwaiti Population. Med Princ Pract.

[31] Jawdat D (2019). HLA-A, B, C, DRB1 and DQB1 allele and haplotype frequencies in volunteer bone marrow donors from Eastern Region of Saudi Arabia. HLA.

[32] Alfraih F (2021). High-resolution HLA allele and haplotype frequencies of the Saudi Arabian population based on 45,457 individuals and corresponding stem cell donor matching probabilities. Hum. Immunol..

[33] Hodgson JA, Mulligan CJ, Al-Meeri A, Raaum RL (2014). Early back-to-Africa migration into the Horn of Africa. PLoS Genet..

[34] Abu-Amero KK, González AM, Larruga JM, Bosley TM, Cabrera VM (2007). Eurasian and African mitochondrial DNA influences in the Saudi Arabian population. BMC Evol. Biol..

[35] Tay GK (2021). Segregation analysis of genotyped and family-phased, long range MHC classical class I and class II haplotypes in 5 families with type 1 diabetes proband in the United Arab Emirates. Front. Genet..

[36] Marsh SGE (2010). An update to HLA Nomenclature, 2010. Bone Marrow Transpl..

[37] Lancaster AK, Single RM, Solberg OD, Nelson MP, Thomson G (2007). PyPop update–a software pipeline for large-scale multilocus population genomics. Tissue Antigens.

[38] Ristow PG, D’Amato ME (2017). Forensic statistics analysis toolbox (FORSTAT): A streamlined workflow for forensic statistics. Forensic Sci. Int. Genet. Suppl. Ser..

[39] Gonzalez-Galarza FF (2020). Allele frequency net database (AFND) 2020 update: Gold-standard data classification, open access genotype data and new query tools. Nucleic Acids Res..

[40] Yasuda N (1988). HLA polymorphism information content (PIC). Jinrui Idengaku Zasshi.

[41] Rodriguez-Reyna TS (2015). HLA Class I and II Blocks Are Associated to Susceptibility, Clinical Subtypes and Autoantibodies in Mexican Systemic Sclerosis (SSc) Patients. PLoS ONE.

[42] AlSafar HS (2019). Introducing the first whole genomes of nationals from the United Arab Emirates. Sci. Rep..

[43] Daw Elbait G, Henschel A, Tay GK, Al Safar HS (2020). Whole genome sequencing of four representatives from the admixed population of the United Arab Emirates. Front Genet.

[44] Aljasmi FA (2020). Genomic landscape of the mitochondrial genome in the United Arab Emirates native population. Genes.

[45] Kulski JK, AlSafar HS, Mawart A, Henschel A, Tay GK (2019). HLA class I allele lineages and haplotype frequencies in Arabs of the United Arab Emirates. Int. J. Immunogenet..

[46] Witt CS (2002). Common HLA-B8-DR3 haplotype in Northern India is different from that found in Europe. Tissue Antigens.

[47] Kaur G (2008). Autoimmune-associated HLA-B8-DR3 haplotypes in Asian Indians are unique in C4 complement gene copy numbers and HSP-2 1267A/G. Hum. Immunol..

[48] Layrisse Z (2001). Extended HLA haplotypes in a carib amerindian population: The Yucpa of the Perija Range. Hum. Immunol..

[49] Cao K (2004). Differentiation between African populations is evidenced by the diversity of alleles and haplotypes of HLA class I loci. Tissue Antigens.

[50] Williams F (2001). Analysis of the distribution of HLA-B alleles in populations from five continents. Hum. Immunol..

[51] Fernandes V (2019). Genome-wide characterization of Arabian Peninsula populations: Shedding light on the history of a fundamental bridge between continents. Mol. Biol. Evol..

[52] Boivin N, Fuller DQ (2009). Shell Middens, ships and seeds: Exploring coastal subsistence, maritime trade and the dispersal of domesticates in and around the ancient Arabian Peninsula. J. World Prehist..

[53] Gostick E (2007). Functional and biophysical characterization of an HLA-A*6801-restricted HIV-specific T cell receptor. Eur. J. Immunol..

[54] Mehra N, Kumar N, Kaur G, Kanga U, Tandon N (2007). Biomarkers of susceptibility to type 1 diabetes with special reference to the Indian population. Indian J. Med. Res..

[55] Robinson J (2020). Ipd-imgt/hla database. Nucleic Acids Res.

[56] Farjadian S (2004). Molecular analysis of HLA allele frequencies and haplotypes in Baloch of Iran compared with related populations of Pakistan. Tissue Antigens.

[57] Cattley SK (2000). Further characterization of MHC haplotypes demonstrates conservation telomeric of HLA-A: Update of the 4AOH and 10IHW cell panels. Eur. J. Immunogenet..

[58] Wallace GR (2014). HLA-B*51 the primary risk in Behçet disease. Proc. Natl. Acad. Sci. U.S.A..

[59] Saylan, T., Mat, C., Fresko, I. & Melikoğlu, M. Behçet's disease in the Middle East. *Clin Dermatol* **17**, 209–223; discussion 105–206, doi:10.1016/s0738-081x(99)00013-9 (1999). 10.1016/s0738-081x(99)00013-9 10330603

[60] Ohno S (1982). Close association of HLA-Bw51 with Behçet's disease. Arch. Ophthalmol..

[61] Verity, D., Wallace, G., Vaughan, R. & Stanford, M. J. B. j. o. o. Behçet’s disease: From Hippocrates to the third millennium. *Br. J. Ophthalmol.* **87**, 1175–1183 (2003). 10.1136/bjo.87.9.1175 PMC1771837 12928293

[62] Heard-Bey F (1982). From Trucial States to United Arab Emirates: A society in Transition.

[63] Kutanan W, Kitpipit T, Phetpeng S, Thanakiatkrai P (2014). Forensic STR loci reveal common genetic ancestry of the Thai-Malay Muslims and Thai Buddhists in the deep Southern region of Thailand. J Hum Genet.

[64] Yao H-B (2016). Genetic evidence for an East Asian origin of Chinese Muslim populations Dongxiang and Hui. Sci. Rep..

[65] Eaaswarkhanth M (2009). Diverse genetic origin of Indian Muslims: Evidence from autosomal STR loci. J. Hum. Genet..

[66] Eaaswarkhanth M (2010). Traces of sub-Saharan and Middle Eastern lineages in Indian Muslim populations. Eur. J. Hum. Genet..

[67] Jones, R. J., Tay, G. K., Mawart, A. & Alsafar, H. J. A. o. h. b. Y-Chromosome haplotypes reveal relationships between populations of the Arabian Peninsula, North Africa and South Asia. *Ann. Hum. Biol.* **44**, 738–746 (2017). 10.1080/03014460.2017.1384508 28948851

[68] Agarwal RK (2017). The case for high resolution extended 6-Loci HLA typing for identifying related donors in the Indian subcontinent. Biol. Blood Marrow Transpl..

[69] Buhler S (2019). High-resolution HLA phased haplotype frequencies to predict the success of unrelated donor searches and clinical outcome following hematopoietic stem cell transplantation. Bone Marrow Transpl..

[70] Fuji S (2015). A single high-resolution HLA mismatch has a similar adverse impact on the outcome of related hematopoietic stem cell transplantation as a single low-resolution HLA mismatch. Am. J. Hematol..

[71] Armstrong AE (2017). The impact of high-resolution HLA-A, HLA-B, HLA-C, and HLA-DRB1 on transplant-related outcomes in single-unit umbilical cord blood transplantation in pediatric patients. J. Pediatric Hematol. Oncol..

[72] Kulski JK, Mawart A, Marie K, Tay GK, AlSafar HS (2019). MHC class I polymorphic Alu insertion (POALIN) allele and haplotype frequencies in the Arabs of the United Arab Emirates and other world populations. Int. J. Immunogenet..

[73] Dunn DS, Tait BD, Kulski JK (2005). The distribution of polymorphic Alu insertions within the MHC class I HLA-B7 and HLA-B57 haplotypes. Immunogenetics.

[74] Kulski JK, Shigenari A, Inoko H (2011). Genetic variation and hitchhiking between structurally polymorphic Alu insertions and HLA-A, -B, and -C alleles and other retroelements within the MHC class I region. Tissue Antigens.

