# Supplementary material for: Characterizing the diversity of MHC conserved extended haplotypes using families from the United Arab Emirates
Source: Sci Rep. 2022 May 3;12:7165. doi: 10.1038/s41598-022-11256-y (PMC9065074; doi:10.1038/s41598-022-11256-y)
Supplement: Supplementary file 1 — Supplementary Tables. [file 41598_2022_11256_MOESM1_ESM.docx]

Table S 1: Study Subjects from the 41 UAE families.

| Family ID | Parents | Offspring | Family ID | Parents | Offspring |
| --- | --- | --- | --- | --- | --- |
| HF1 | 2 | 4 | HF30 | 2 | 3 |
| HF2 | 2 | 4 | HF31 | 2 | 3 |
| HF4 | 2 | 3 | HF32 | 2 | 1 |
| HF6 | 2 | 4 | HF36 | 2 | 5 |
| HF7 | 2 | 3 | HF8 | 5 | 11 |
| HF9 | 2 | 3 | HF38 | 2 | 1 |
| HF10 | 2 | 4 | HF39 | 2 | 3 |
| HF11 | 2 | 3 | DF13 | 2 | 1 |
| HF12 | 2 | 2 | DF26 | 2 | 2 |
| HF16 | 2 | 2 | DF28 | 2 | 2 |
| HF17 | 2 | 2 | DF31 | 2 | 2 |
| HF18 | 1 | 5 | DF35 | 2 | 1 |
| HF19 | 2 | 6 | DF37 | 2 | 15 |
| HF20 | 2 | 5 | DF2 | 2 | 2 |
| HF22 | 2 | 5 | DF14 | 2 | 3 |
| HF24 | 2 | 10 | DF22 | 2 | 1 |
| HF25 | 2 | 9 | DF23 | 2 | 1 |
| HF26 | 2 | 6 | DF24 | 2 | 3 |
| HF27 | 2 | 3 | DF4 | 2 | 1 |
| HF28 | 2 | 2 | DF12 | 1 | 3 |
| HF29 | 2 | 3 | ***Total: 41*** | ***83*** | ***152*** |

Table S 2: Guo and Thompson Hardy-Weinberg expectation, polymorphic information capacity (PIC) and Power of Discrimination (PD) calculated for 6 HLA. Obs. Het.: observed heterozygosity; Exp. Het.: expected heterozygosity; * Significant deviation from HWE.

| **Locus** | **Obs. Het.** | **Exp. Het.** | **P-Value** | **PIC** | **PD** |
| --- | --- | --- | --- | --- | --- |
| A | 72 | 78.96 | 0.2340 | 0.9257 | 0.9821 |
| C | 79 | 77.24 | 0.7352 | 0.9022 | 0.9755 |
| B | 78 | 80.43 | 0.1719 | 0.9437 | 0.9838 |
| DRB1 | 73 | 74.98 | 0.3297 | 0.8756 | 0.9721 |
| DQA1 | 68 | 69.97 | 0.1131 | 0.8186 | 0.9461 |
| DQB1 | 62 | 70.41 | 0.0037* | 0.8256 | 0.9456 |

Table S 3: Ewens-Watterson (EW) homozygosity test of selective neutrality for 6 HLA loci. Fnd: normalized deviation of homozygosity (F).

| **Locus** | **observed F** | **expected F** | **variance in F** | **(Fnd)** | **P-value** |
| --- | --- | --- | --- | --- | --- |
| A | 0.071 | 0.0839 | 0.0005 | -0.5624 | 0.3077 |
| C | 0.0913 | 0.0952 | 0.0007 | -0.1439 | 0.5487 |
| B | 0.0538 | 0.0605 | 0.0002 | -0.4707 | 0.3579 |
| DRB1 | 0.1179 | 0.0875 | 0.0006 | 1.2528 | 0.9024 |
| DQA1 | 0.1670 | 0.2362 | 0.0071 | -0.8219 | 0.1849 |
| DQB1 | 0.1618 | 0.1904 | 0.0044 | -0.4339 | 0.3956 |

Table S 4: HLA -C-B two-locus haplotype counts observed in the UAE cohort.

| Haplotypes | | | | Count (n=170) | Haplotypes | | | | Count (n=170) |
| --- | --- | --- | --- | --- | --- | --- | --- | --- | --- |
| HLA- C* | 07:02 | -B* | 08:01 | 16 | HLA- C* | 03:04 | -B* | 40:06 | 1 |
| HLA- C* | 15:02 | -B* | 40:06 | 12 | HLA- C* | 04:01 | -B* | 15:10 | 1 |
| HLA- C* | 06:02 | -B* | 50:01 | 10 | HLA- C* | 04:01 | -B* | 15:22 | 1 |
| HLA- C* | 03:02 | -B* | 58:01 | 8 | HLA- C* | 04:01 | -B* | 18:01 | 1 |
| HLA- C* | 04:01 | -B* | 35:03 | 8 | HLA- C* | 04:01 | -B* | 50:01 | 1 |
| HLA- C* | 04:01 | -B* | 35:01 | 7 | HLA- C* | 04:03 | -B* | 13:01 | 1 |
| HLA- C* | 16:02 | -B* | 51:01 | 7 | HLA- C* | 06:02 | -B* | 13:02 | 1 |
| HLA- C* | 04:01 | -B* | 35:08 | 5 | HLA- C* | 06:02 | -B* | 37:01 | 1 |
| HLA- C* | 12:02 | -B* | 52:01 | 5 | HLA- C* | 06:02 | -B* | 41:01 | 1 |
| HLA- C* | 15:02 | -B* | 51:01 | 5 | HLA- C* | 06:02 | -B* | 45:01 | 1 |
| HLA- C* | 04:01 | -B* | 53:01 | 4 | HLA- C* | 06:02 | -B* | 53:01 | 1 |
| HLA- C* | 07:01 | -B* | 18:01 | 4 | HLA- C* | 07:01 | -B* | 15:17 | 1 |
| HLA- C* | 07:02 | -B* | 07:02 | 4 | HLA- C* | 07:01 | -B* | 57:01 | 1 |
| HLA- C* | 08:02 | -B* | 14:02 | 4 | HLA- C* | 07:02 | -B* | 18:01 | 1 |
| HLA- C* | 17:01 | -B* | 42:01 | 4 | HLA- C* | 07:04 | -B* | 18:01 | 1 |
| HLA- C* | 06:02 | -B* | 57:01 | 3 | HLA- C* | 07:04 | -B* | 44:02 | 1 |
| HLA- C* | 12:03 | -B* | 38:01 | 3 | HLA- C* | 07:18 | -B* | 47:03 | 1 |
| HLA- C* | 04:01 | -B* | 35:02 | 2 | HLA- C* | 08:01 | -B* | 15:02 | 1 |
| HLA- C* | 04:01 | -B* | 51:01 | 2 | HLA- C* | 08:02 | -B* | 40:16 | 1 |
| HLA- C* | 06:02 | -B* | 58:02 | 2 | HLA- C* | 12:03 | -B* | 13:01 | 1 |
| HLA- C* | 07:01 | -B* | 08:01 | 2 | HLA- C* | 12:03 | -B* | 18:01 | 1 |
| HLA- C* | 07:01 | -B* | 41:01 | 2 | HLA- C* | 12:03 | -B* | 58:01 | 1 |
| HLA- C* | 15:04 | -B* | 51:01 | 2 | HLA- C* | 14:02 | -B* | 15:67 | 1 |
| HLA- C* | 15:05 | -B* | 07:05 | 2 | HLA- C* | 14:02 | -B* | 51:01 | 1 |
| HLA- C* | 15:13 | -B* | 51:01 | 2 | HLA- C* | 15:02 | -B* | 45:01 | 1 |
| HLA- C* | 16:02 | -B* | 39:01 | 2 | HLA- C* | 15:05 | -B* | 27:03 | 1 |
| HLA- C* | 01:02 | -B* | 53:01 | 1 | HLA- C* | 15:05 | -B* | 73:01 | 1 |
| HLA- C* | 01:02 | -B* | 55:01 | 1 | HLA- C* | 12:194 | -B* | 18:01 | 1 |
| HLA- C* | 02:02 | -B* | 18:01 | 1 | HLA- C* | 16:01 | -B* | 44:03 | 1 |
| HLA- C* | 02:02 | -B* | 27:03 | 1 | HLA- C* | 16:01 | -B* | 45:01 | 1 |
| HLA- C* | 02:10 | -B* | 14:01 | 1 | HLA- C* | 16:02 | -B* | 51:08 | 1 |
| HLA- C* | 02:16 | -B* | 15:03 | 1 | HLA- C* | 16:04 | -B* | 44:02 | 1 |
| HLA- C* | 03:02 | -B* | 35:01 | 1 | HLA- C* | 17:01 | -B* | 41:01 | 1 |
| HLA- C* | 03:03 | -B* | 51:01 | 1 | HLA- C* | 18:01 | -B* | 81:01 | 1 |
| HLA- C* | 03:04 | -B* | 15:10 | 1 |  |  |  |  |  |

Table S 5: HLA -DRB1-DQA1-DQB1 three-locus haplotype counts observed in the UAE cohort. NA#: missing allele due to sequencing error.

| Haplotype | | | | | | Count (n=170) | Haplotype | | | | | | Count (n=170) |
| --- | --- | --- | --- | --- | --- | --- | --- | --- | --- | --- | --- | --- | --- |
| HLA-DRB1* | 03:01 | HLA-DRB1* | 05:01 | -DQB1* | 02:01 | 43 | HLA-DRB1* | 04:05 | HLA-DRB1* | 03:01 | -DQB1* | 03:02 | 1 |
| HLA-DRB1* | 16:01 | HLA-DRB1* | 01:02 | -DQB1* | 05:02 | 15 | HLA-DRB1* | 04:05 | HLA-DRB1* | 03:03 | -DQB1* | 02:02 | 1 |
| HLA-DRB1* | 16:02 | HLA-DRB1* | 01:02 | -DQB1* | 05:02 | 14 | HLA-DRB1* | 04:05 | HLA-DRB1* | 03:03 | -DQB1* | 05:02 | 1 |
| HLA-DRB1* | 11:01 | HLA-DRB1* | 05:05 | -DQB1* | 03:01 | 7 | HLA-DRB1* | 07:01 | HLA-DRB1* | 01:02 | -DQB1* | 02:02 | 1 |
| HLA-DRB1* | 04:05 | HLA-DRB1* | 03:03 | -DQB1* | 03:02 | 6 | HLA-DRB1* | 07:01 | HLA-DRB1* | 02:01 | -DQB1* | 02:01 | 1 |
| HLA-DRB1* | 04:02 | HLA-DRB1* | 03:01 | -DQB1* | 03:02 | 5 | HLA-DRB1* | 07:01 | HLA-DRB1* | 02:01 | -DQB1* | 04:02 | 1 |
| HLA-DRB1* | 07:01 | HLA-DRB1* | 02:01 | -DQB1* | 02:02 | 5 | HLA-DRB1* | 07:01 | HLA-DRB1* | 03:03 | -DQB1* | 02:02 | 1 |
| HLA-DRB1* | 01:01 | HLA-DRB1* | 01:01 | -DQB1* | 05:01 | 4 | HLA-DRB1* | 08:04 | HLA-DRB1* | 04:01 | -DQB1* | 04:02 | 1 |
| HLA-DRB1* | 01:02 | HLA-DRB1* | 01:01 | -DQB1* | 05:01 | 4 | HLA-DRB1* | 09:01 | HLA-DRB1* | 03:02 | -DQB1* | 03:02 | 1 |
| HLA-DRB1* | 11:04 | HLA-DRB1* | 05:05 | -DQB1* | 03:01 | 4 | HLA-DRB1* | 09:01 | HLA-DRB1* | 03:03 | -DQB1* | 02:01 | 1 |
| HLA-DRB1* | 10:01 | HLA-DRB1* | 01:05 | -DQB1* | 05:01 | 3 | HLA-DRB1* | 11:01 | HLA-DRB1* | 01:02 | -DQB1* | 05:02 | 1 |
| HLA-DRB1* | 15:02 | HLA-DRB1* | 01:03 | -DQB1* | 06:01 | 3 | HLA-DRB1* | 11:01 | HLA-DRB1* | 03:02 | -DQB1* | 03:35 | 1 |
| HLA-DRB1* | 15:03 | HLA-DRB1* | 01:02 | -DQB1* | 06:02 | 3 | HLA-DRB1* | 11:01 | HLA-DRB1* | 05:05 | -DQB1* | 02:01 | 1 |
| HLA-DRB1* | 03:01 | HLA-DRB1* | 05:01 | -DQB1* | 02:02 | 2 | HLA-DRB1* | 11:01 | HLA-DRB1* | 05:05 | -DQB1* | 03:27 | 1 |
| HLA-DRB1* | 03:02 | HLA-DRB1* | 04:01 | -DQB1* | 04:02 | 2 | HLA-DRB1* | 11:01 | HLA-DRB1* | 05:09 | -DQB1* | 05:02 | 1 |
| HLA-DRB1* | 04:06 | HLA-DRB1* | 03:03 | -DQB1* | 04:02 | 2 | HLA-DRB1* | 11:02 | HLA-DRB1* | 05:05 | -DQB1* | 03:19 | 1 |
| HLA-DRB1* | 15:01 | HLA-DRB1* | 01:02 | -DQB1* | 06:01 | 2 | HLA-DRB1* | 11:04 | HLA-DRB1* | NA | -DQB1* | 05:01 | 1 |
| HLA-DRB1* | 15:01 | HLA-DRB1* | 01:02 | -DQB1* | 06:02 | 2 | HLA-DRB1* | 13:01 | HLA-DRB1* | 01:03 | -DQB1* | 06:02 | 1 |
| HLA-DRB1* | 01:01 | HLA-DRB1* | 01:01 | -DQB1* | NA | 1 | HLA-DRB1* | 13:01 | HLA-DRB1* | 01:03 | -DQB1* | 06:03 | 1 |
| HLA-DRB1* | 01:01 | HLA-DRB1* | 01:03 | -DQB1* | 05:01 | 1 | HLA-DRB1* | 13:02 | HLA-DRB1* | 01:02 | -DQB1* | 06:04 | 1 |
| HLA-DRB1* | 01:02 | HLA-DRB1* | 01:01 | -DQB1* | 03:02 | 1 | HLA-DRB1* | 13:03 | HLA-DRB1* | 05:05 | -DQB1* | 03:01 | 1 |
| HLA-DRB1* | 03:01 | HLA-DRB1* | 01:02 | -DQB1* | 05:02 | 1 | HLA-DRB1* | 14:04 | HLA-DRB1* | 01:04 | -DQB1* | 05:03 | 1 |
| HLA-DRB1* | 03:01 | HLA-DRB1* | 01:03 | -DQB1* | 06:01 | 1 | HLA-DRB1* | 14:15 | HLA-DRB1* | 01:04 | -DQB1* | 05:03 | 1 |
| HLA-DRB1* | 03:01 | HLA-DRB1* | 05:01 | -DQB1* | 06:01 | 1 | HLA-DRB1* | 14:21 | HLA-DRB1* | 01:04 | -DQB1* | 05:03 | 1 |
| HLA-DRB1* | 03:01 | HLA-DRB1* | 05:01 | -DQB1* | NA | 1 | HLA-DRB1* | 15:01 | HLA-DRB1* | 01:03 | -DQB1* | 06:01 | 1 |
| HLA-DRB1* | 03:07 | HLA-DRB1* | 01:02 | -DQB1* | 05:02 | 1 | HLA-DRB1* | 15:02 | HLA-DRB1* | 01:03 | -DQB1* | 02:01 | 1 |
| HLA-DRB1* | 04:01 | HLA-DRB1* | 03:01 | -DQB1* | 03:02 | 1 | HLA-DRB1* | 15:03 | HLA-DRB1* | 05:05 | -DQB1* | 03:01 | 1 |
| HLA-DRB1* | 04:02 | HLA-DRB1* | 01:01 | -DQB1* | 03:02 | 1 | HLA-DRB1* | 15:06 | HLA-DRB1* | 01:05 | -DQB1* | 05:02 | 1 |
| HLA-DRB1* | 04:03 | HLA-DRB1* | 03:01 | -DQB1* | 02:01 | 1 | HLA-DRB1* | 16:02 | HLA-DRB1* | 01:02 | -DQB1* | NA | 1 |
| HLA-DRB1* | 04:03 | HLA-DRB1* | 03:01 | -DQB1* | 03:02 | 1 | HLA-DRB1* | 16:02 | HLA-DRB1* | 05:01 | -DQB1* | 02:01 | 1 |
| HLA-DRB1* | 04:04 | HLA-DRB1* | 03:01 | -DQB1* | 03:02 | 1 | HLA-DRB1* | 01:02 | HLA-DRB1* | 03:01 | -DQB1* | 05:01 | 1 |

Table S 6: HLA- DPA1-DPB1 two-locus haplotype counts observed in the UAE cohort.

| Haplotypes | | | | Count (n=170) |
| --- | --- | --- | --- | --- |
| HLA-DPA1* | 01:03 | -DPB1* | 04:01 | 47 |
| HLA-DPA1* | 01:03 | -DPB1* | 02:01 | 32 |
| HLA-DPA1* | 02:01 | -DPB1* | 14:01 | 15 |
| HLA-DPA1* | 01:03 | -DPB1* | 04:02 | 14 |
| HLA-DPA1* | 01:03 | -DPB1* | 104:01 | 6 |
| HLA-DPA1* | 02:01 | -DPB1* | 17:01 | 6 |
| HLA-DPA1* | 01:03 | -DPB1* | 03:01 | 5 |
| HLA-DPA1* | 01:03 | -DPB1* | 18:01 | 4 |
| HLA-DPA1* | 02:01 | -DPB1* | 10:01 | 4 |
| HLA-DPA1* | 02:01 | -DPB1* | 13:01 | 4 |
| HLA-DPA1* | 02:01 | -DPB1* | 01:01 | 3 |
| HLA-DPA1* | 02:01 | -DPB1* | 09:01 | 3 |
| HLA-DPA1* | 02:02 | -DPB1* | 01:01 | 3 |
| HLA-DPA1* | 02:07 | -DPB1* | 04:01 | 3 |
| HLA-DPA1* | 01:03 | -DPB1* | 14:01 | 2 |
| HLA-DPA1* | 02:01 | -DPB1* | 107:01 | 2 |
| HLA-DPA1* | 02:02 | -DPB1* | 03:01 | 2 |
| HLA-DPA1* | 03:01 | -DPB1* | 105:01 | 2 |
| HLA-DPA1* | 01:03 | -DPB1* | 01:01 | 1 |
| HLA-DPA1* | 01:03 | -DPB1* | 13:01 | 1 |
| HLA-DPA1* | 01:03 | -DPB1* | 45:01 | 1 |
| HLA-DPA1* | 01:03 | -DPB1* | 124:01 | 1 |
| HLA-DPA1* | 01:04 | -DPB1* | 15:01 | 1 |
| HLA-DPA1* | 01:14 | -DPB1* | 04:01 | 1 |
| HLA-DPA1* | 02:01 | -DPB1* | 02:01 | 1 |
| HLA-DPA1* | 02:01 | -DPB1* | 04:01 | 1 |
| HLA-DPA1* | 02:01 | -DPB1* | 26:01 | 1 |
| HLA-DPA1* | 02:01 | -DPB1* | 39:01 | 1 |
| HLA-DPA1* | 02:01 | -DPB1* | 91:01 | 1 |
| HLA-DPA1* | 02:02 | -DPB1* | 13:01 | 1 |
| HLA-DPA1* | 02:09 | -DPB1* | 02:01 | 1 |

Table S 7: Abbreviations and references of AFDN datasets used in the construction of the PCA plot and phylogenetic tree. The current cohort is denoted by ‘UAE’.

| Abbreviation | Dataset | Sample size | Reference | Abbreviation | Dataset | Sample size | Reference |
| --- | --- | --- | --- | --- | --- | --- | --- |
| Bra | Brazil Mixed | 108 | ^1^ | JpC | Japan Central | 371 | ^2^ |
| Cam | Cameroon Beti | 174 | ^3^ | Jor | Jordan Amman | 146 | ^1^ |
| ChM | Chile Mapuche | 66 | ^4^ | Key | Kenya | 144 | ^1^ |
| ChN | China Hubei Han | 3732 | ^5^ | KeL | Kenya Luo | 265 | ^1^ |
| ChZ | China Zhejiang Han | 1734 | ^1^ | KeN | Kenya Nandi | 240 | ^1^ |
| Col | Colombia Bogotá Cord Blood | 1463 | ^6^ | Mal | Malaysia Peninsular Malay | 951 | ^7^ |
| Cos | Costa Rica Central Valley Mestizo | 221 | ^8^ | Net | Netherlands Leiden | 1305 | ^1^ |
| CoG | Costa Rica Guanacaste Mestizo | 110 | ^8^ | Oma | Oman | 118 | ^1^ |
| Cro | Croatia pop 4 | 4000 | ^9^ | PaB | Pakistan Baloch | 66 | ^10^ |
| Cze | Czech Republic NMDR | 5099 | ^1^ | PkB | Pakistan Brahui | 104 | ^10^ |
| Fin | Finland | 91 | ^1^ | Pan | Panama | 462 | ^11^ |
| Ger | Germany pop 8 | 39689 | ^1^ | Phi | Philippines Ivatan | 50 | ^1^ |
| Gha | Ghana Ga-Adangbe | 131 | ^12^ | Pol | Poland DKMS | 20653 | ^13^ |
| HKC | Hong Kong Chinese BMDR | 7595 | ^1^ | Sgh | Saudi Arabia Guraiat and Hail | 213 | ^1^ |
| InK | India Kerala Malayalam speaking | 356 | ^14^ | Sen | Senegal Niokholo Mandenka | 165 | ^1^ |
| Ino | India North pop2 | 72 | ^15^ | SoK | South Korea pop 10 | 4128 | ^16^ |
| Int | India Tamil Nadu | 2492 | ^17^ | SK3 | South Korea pop 3 | 485 | ^18^ |
| Ind | Indonesia Sundanese and Javanese | 201 | ^1^ | Spn | Spain (Catalunya, Navarra, Extremadura, Aaragón, Cantabria | 4335 | ^19^ |
| IrB | Iran Balouch | 100 | ^1^ | Sud | Sudan Mixed | 200 | ^20^ |
| Ire | Ireland Northern | 1000 | ^21^ | Tun | Tunisia Gabes | 95 | ^22^ |
| IrS | Ireland South | 250 | ^1^ | UAE | UAE | 85 | Current cohort |
| IsA | Israel Arab pop 2 | 12301 | ^1^ | UgK | Uganda Kampala | 161 | ^1^ |
| IsJ | Israel Ashkenazi Jews pop 3 | 4625 | ^1^ | Uk2 | Uganda Kampala pop 2 | 175 | ^1^ |
| It3 | Italy North pop 3 | 97 | ^1^ | Zim | Zimbabwe Harare Shona | 230 | ^1^ |
| It5 | Italy pop 5 | 975 | ^23^ | SwN | Sweden Northern Sami | 154 | ^24^ |

Table S 8: Complete list of phase-segregated 5-locus haplotypes in this study. NA: missing allele due to sequencing error.

| Haplotypes | | | | | | | | | | n (N=170) | Haplotypes | | | | | | | | | | n (N=170) |
| --- | --- | --- | --- | --- | --- | --- | --- | --- | --- | --- | --- | --- | --- | --- | --- | --- | --- | --- | --- | --- | --- |
| HLA- C* | 07:02 | -B* | 08:01 | -DRB1* | 03:01 | -DQA1* | 05:01 | -DQB1* | 02:01 | 16 | HLA- C* | 06:02 | -B* | 57:01 | -DRB1* | 13:01 | -DQA1* | 01:03 | -DQB1* | 06:03 | 1 |
| HLA- C* | 15:02 | -B* | 40:06 | -DRB1* | 16:02 | -DQA1* | 01:02 | -DQB1* | 05:02 | 6 | HLA- C* | 06:02 | -B* | 57:01 | -DRB1* | 16:02 | -DQA1* | 01:02 | -DQB1* | 05:02 | 1 |
| HLA- C* | 16:02 | -B* | 51:01 | -DRB1* | 16:01 | -DQA1* | 01:02 | -DQB1* | 05:02 | 5 | HLA- C* | 06:02 | -B* | 58:02 | -DRB1* | 03:02 | -DQA1* | 04:01 | -DQB1* | 04:02 | 1 |
| HLA- C* | 03:02 | -B* | 58:01 | -DRB1* | 03:01 | -DQA1* | 05:01 | -DQB1* | 02:01 | 4 | HLA- C* | 06:02 | -B* | 58:02 | -DRB1* | 04:05 | -DQA1* | 03:01 | -DQB1* | 03:02 | 1 |
| HLA- C* | 03:02 | -B* | 58:01 | -DRB1* | 16:01 | -DQA1* | 01:02 | -DQB1* | 05:02 | 4 | HLA- C* | 07:01 | -B* | 08:01 | -DRB1* | 03:01 | -DQA1* | 01:03 | -DQB1* | 06:01 | 1 |
| HLA- C* | 04:01 | -B* | 35:08 | -DRB1* | 11:04 | -DQA1* | 05:05 | -DQB1* | 03:01 | 3 | HLA- C* | 07:01 | -B* | 08:01 | -DRB1* | 03:01 | -DQA1* | 05:01 | -DQB1* | 02:01 | 1 |
| HLA- C* | 06:02 | -B* | 50:01 | -DRB1* | 03:01 | -DQA1* | 05:01 | -DQB1* | 02:01 | 3 | HLA- C* | 07:01 | -B* | 15:17 | -DRB1* | 11:01 | -DQA1* | 05:05 | -DQB1* | 03:01 | 1 |
| HLA- C* | 07:02 | -B* | 07:02 | -DRB1* | 03:01 | -DQA1* | 05:01 | -DQB1* | 02:01 | 3 | HLA- C* | 07:01 | -B* | 18:01 | -DRB1* | 11:01 | -DQA1* | 05:05 | -DQB1* | 03:27 | 1 |
| HLA- C* | 04:01 | -B* | 35:01 | -DRB1* | 01:01 | -DQA1* | 01:01 | -DQB1* | 05:01 | 2 | HLA- C* | 07:01 | -B* | 18:01 | -DRB1* | 15:01 | -DQA1* | 01:02 | -DQB1* | 06:01 | 1 |
| HLA- C* | 04:01 | -B* | 35:03 | -DRB1* | 04:05 | -DQA1* | 03:03 | -DQB1* | 03:02 | 2 | HLA- C* | 07:01 | -B* | 57:01 | -DRB1* | 15:01 | -DQA1* | 01:02 | -DQB1* | 06:02 | 1 |
| HLA- C* | 04:01 | -B* | 53:01 | -DRB1* | 03:01 | -DQA1* | 05:01 | -DQB1* | 02:01 | 2 | HLA- C* | 07:02 | -B* | 07:02 | -DRB1* | 03:01 | -DQA1* | 05:01 | -DQB1* | NA | 1 |
| HLA- C* | 04:01 | -B* | 53:01 | -DRB1* | 04:05 | -DQA1* | 03:03 | -DQB1* | 03:02 | 2 | HLA- C* | 07:02 | -B* | 18:01 | -DRB1* | 16:02 | -DQA1* | 05:01 | -DQB1* | 02:01 | 1 |
| HLA- C* | 06:02 | -B* | 50:01 | -DRB1* | 04:06 | -DQA1* | 03:03 | -DQB1* | 04:02 | 2 | HLA- C* | 07:04 | -B* | 18:01 | -DRB1* | 16:01 | -DQA1* | 01:02 | -DQB1* | 05:02 | 1 |
| HLA- C* | 06:02 | -B* | 50:01 | -DRB1* | 07:01 | -DQA1* | 02:01 | -DQB1* | 02:02 | 2 | HLA- C* | 07:04 | -B* | 44:02 | -DRB1* | 11:01 | -DQA1* | 05:05 | -DQB1* | 03:01 | 1 |
| HLA- C* | 07:01 | -B* | 18:01 | -DRB1* | 03:01 | -DQA1* | 05:01 | -DQB1* | 02:01 | 2 | HLA- C* | 07:18 | -B* | 47:03 | -DRB1* | 03:01 | -DQA1* | 05:01 | -DQB1* | 06:01 | 1 |
| HLA- C* | 07:01 | -B* | 41:01 | -DRB1* | 04:05 | -DQA1* | 03:03 | -DQB1* | 03:02 | 2 | HLA- C* | 08:01 | -B* | 15:02 | -DRB1* | 15:01 | -DQA1* | 01:02 | -DQB1* | 06:01 | 1 |
| HLA- C* | 08:02 | -B* | 14:02 | -DRB1* | 03:01 | -DQA1* | 05:01 | -DQB1* | 02:01 | 2 | HLA- C* | 08:02 | -B* | 14:02 | -DRB1* | 01:02 | -DQA1* | 01:01 | -DQB1* | 05:01 | 1 |
| HLA- C* | 12:02 | -B* | 52:01 | -DRB1* | 15:02 | -DQA1* | 01:03 | -DQB1* | 06:01 | 2 | HLA- C* | 08:02 | -B* | 14:02 | -DRB1* | 03:01 | -DQA1* | 01:02 | -DQB1* | 05:02 | 1 |
| HLA- C* | 15:04 | -B* | 51:01 | -DRB1* | 11:01 | -DQA1* | 05:05 | -DQB1* | 03:01 | 2 | HLA- C* | 08:02 | -B* | 40:16 | -DRB1* | 07:01 | -DQA1* | 03:03 | -DQB1* | 02:02 | 1 |
| HLA- C* | 16:02 | -B* | 39:01 | -DRB1* | 16:01 | -DQA1* | 01:02 | -DQB1* | 05:02 | 2 | HLA- C* | 12:02 | -B* | 52:01 | -DRB1* | 03:01 | -DQA1* | 05:01 | -DQB1* | 02:01 | 1 |
| HLA- C* | 01:02 | -B* | 53:01 | -DRB1* | 03:01 | -DQA1* | 05:01 | -DQB1* | 02:01 | 1 | HLA- C* | 12:02 | -B* | 52:01 | -DRB1* | 15:02 | -DQA1* | 01:03 | -DQB1* | 02:01 | 1 |
| HLA- C* | 01:02 | -B* | 55:01 | -DRB1* | 14:21 | -DQA1* | 01:04 | -DQB1* | 05:03 | 1 | HLA- C* | 12:02 | -B* | 52:01 | -DRB1* | 16:02 | -DQA1* | 01:02 | -DQB1* | 05:02 | 1 |
| HLA- C* | 02:02 | -B* | 18:01 | -DRB1* | 16:01 | -DQA1* | 01:02 | -DQB1* | 05:02 | 1 | HLA- C* | 12:03 | -B* | 13:01 | -DRB1* | 15:02 | -DQA1* | 01:03 | -DQB1* | 06:01 | 1 |
| HLA- C* | 02:02 | -B* | 27:03 | -DRB1* | 08:04 | -DQA1* | 04:01 | -DQB1* | 04:02 | 1 | HLA- C* | 12:03 | -B* | 18:01 | -DRB1* | 16:02 | -DQA1* | 01:02 | -DQB1* | 05:02 | 1 |
| HLA- C* | 02:10 | -B* | 14:01 | -DRB1* | 03:01 | -DQA1* | 05:01 | -DQB1* | 02:01 | 1 | HLA- C* | 12:03 | -B* | 38:01 | -DRB1* | 04:01 | -DQA1* | 03:01 | -DQB1* | 03:02 | 1 |
| HLA- C* | 02:16 | -B* | 15:03 | -DRB1* | 03:01 | -DQA1* | 05:01 | -DQB1* | 02:02 | 1 | HLA- C* | 12:03 | -B* | 38:01 | -DRB1* | 10:01 | -DQA1* | 01:05 | -DQB1* | 05:01 | 1 |
| HLA- C* | 03:02 | -B* | 35:01 | -DRB1* | 03:01 | -DQA1* | 05:01 | -DQB1* | 02:01 | 1 | HLA- C* | 12:03 | -B* | 38:01 | -DRB1* | 13:01 | -DQA1* | 01:03 | -DQB1* | 06:02 | 1 |
| HLA- C* | 03:03 | -B* | 51:01 | -DRB1* | 03:01 | -DQA1* | 05:01 | -DQB1* | 02:01 | 1 | HLA- C* | 12:03 | -B* | 58:01 | -DRB1* | 04:03 | -DQA1* | 03:01 | -DQB1* | 02:01 | 1 |
| HLA- C* | 03:04 | -B* | 15:10 | -DRB1* | 03:01 | -DQA1* | 05:01 | -DQB1* | 02:01 | 1 | HLA- C* | 12:194 | -B* | 18:01 | -DRB1* | 11:04 | -DQA1* | 05:05 | -DQB1* | 03:01 | 1 |
| HLA- C* | 03:04 | -B* | 40:06 | -DRB1* | 16:02 | -DQA1* | 01:02 | -DQB1* | 05:02 | 1 | HLA- C* | 14:02 | -B* | 15:67 | -DRB1* | 15:03 | -DQA1* | 01:02 | -DQB1* | 06:02 | 1 |

(Continued)

| Haplotypes | | | | | | | | | | n (N=170) | Haplotypes | | | | | | | | | | n (N=170) |
| --- | --- | --- | --- | --- | --- | --- | --- | --- | --- | --- | --- | --- | --- | --- | --- | --- | --- | --- | --- | --- | --- |
| HLA- C* | 04:01 | -B* | 15:10 | -DRB1* | 07:01 | -DQA1* | 02:01 | -DQB1* | 02:02 | 1 | HLA- C* | 14:02 | -B* | 51:01 | -DRB1* | 16:02 | -DQA1* | 01:02 | -DQB1* | NA | 1 |
| HLA- C* | 04:01 | -B* | 15:22 | -DRB1* | 04:05 | -DQA1* | 03:03 | -DQB1* | 02:02 | 1 | HLA- C* | 15:02 | -B* | 40:06 | -DRB1* | 03:01 | -DQA1* | 05:01 | -DQB1* | 02:01 | 1 |
| HLA- C* | 04:01 | -B* | 18:01 | -DRB1* | 11:01 | -DQA1* | 03:02 | -DQB1* | 03:35 | 1 | HLA- C* | 15:02 | -B* | 40:06 | -DRB1* | 10:01 | -DQA1* | 01:05 | -DQB1* | 05:01 | 1 |
| HLA- C* | 04:01 | -B* | 35:01 | -DRB1* | 03:01 | -DQA1* | 05:01 | -DQB1* | 02:02 | 1 | HLA- C* | 15:02 | -B* | 40:06 | -DRB1* | 11:01 | -DQA1* | 01:02 | -DQB1* | 05:02 | 1 |
| HLA- C* | 04:01 | -B* | 35:01 | -DRB1* | 04:02 | -DQA1* | 03:01 | -DQB1* | 03:02 | 1 | HLA- C* | 15:02 | -B* | 40:06 | -DRB1* | 14:04 | -DQA1* | 01:04 | -DQB1* | 05:03 | 1 |
| HLA- C* | 04:01 | -B* | 35:01 | -DRB1* | 11:01 | -DQA1* | 05:05 | -DQB1* | 03:01 | 1 | HLA- C* | 15:02 | -B* | 40:06 | -DRB1* | 15:01 | -DQA1* | 01:03 | -DQB1* | 06:01 | 1 |
| HLA- C* | 04:01 | -B* | 35:01 | -DRB1* | 16:01 | -DQA1* | 01:02 | -DQB1* | 05:02 | 1 | HLA- C* | 15:02 | -B* | 40:06 | -DRB1* | 16:01 | -DQA1* | 01:02 | -DQB1* | 05:02 | 1 |
| HLA- C* | 04:01 | -B* | 35:01 | -DRB1* | 16:02 | -DQA1* | 01:02 | -DQB1* | 05:02 | 1 | HLA- C* | 15:02 | -B* | 45:01 | -DRB1* | 01:02 | -DQA1* | 01:01 | -DQB1* | 03:02 | 1 |
| HLA- C* | 04:01 | -B* | 35:02 | -DRB1* | 11:04 | -DQA1* | NA | -DQB1* | 05:01 | 1 | HLA- C* | 15:02 | -B* | 51:01 | -DRB1* | 01:01 | -DQA1* | 01:01 | -DQB1* | 05:01 | 1 |
| HLA- C* | 04:01 | -B* | 35:02 | -DRB1* | 15:03 | -DQA1* | 05:05 | -DQB1* | 03:01 | 1 | HLA- C* | 15:02 | -B* | 51:01 | -DRB1* | 01:01 | -DQA1* | 01:01 | -DQB1* | NA | 1 |
| HLA- C* | 04:01 | -B* | 35:03 | -DRB1* | 01:01 | -DQA1* | 01:01 | -DQB1* | 05:01 | 1 | HLA- C* | 15:02 | -B* | 51:01 | -DRB1* | 01:01 | -DQA1* | 01:03 | -DQB1* | 05:01 | 1 |
| HLA- C* | 04:01 | -B* | 35:03 | -DRB1* | 03:01 | -DQA1* | 05:01 | -DQB1* | 02:01 | 1 | HLA- C* | 15:02 | -B* | 51:01 | -DRB1* | 11:01 | -DQA1* | 05:05 | -DQB1* | 03:01 | 1 |
| HLA- C* | 04:01 | -B* | 35:03 | -DRB1* | 04:05 | -DQA1* | 03:03 | -DQB1* | 05:02 | 1 | HLA- C* | 15:02 | -B* | 51:01 | -DRB1* | 15:01 | -DQA1* | 01:02 | -DQB1* | 06:02 | 1 |
| HLA- C* | 04:01 | -B* | 35:03 | -DRB1* | 11:01 | -DQA1* | 05:09 | -DQB1* | 05:02 | 1 | HLA- C* | 15:05 | -B* | 07:05 | -DRB1* | 09:01 | -DQA1* | 03:02 | -DQB1* | 03:02 | 1 |
| HLA- C* | 04:01 | -B* | 35:03 | -DRB1* | 14:15 | -DQA1* | 01:04 | -DQB1* | 05:03 | 1 | HLA- C* | 15:05 | -B* | 07:05 | -DRB1* | 10:01 | -DQA1* | 01:05 | -DQB1* | 05:01 | 1 |
| HLA- C* | 04:01 | -B* | 35:03 | -DRB1* | 16:02 | -DQA1* | 01:02 | -DQB1* | 05:02 | 1 | HLA- C* | 15:05 | -B* | 27:03 | -DRB1* | 15:03 | -DQA1* | 01:02 | -DQB1* | 06:02 | 1 |
| HLA- C* | 04:01 | -B* | 35:08 | -DRB1* | 11:01 | -DQA1* | 05:05 | -DQB1* | 03:01 | 1 | HLA- C* | 15:05 | -B* | 73:01 | -DRB1* | 01:02 | -DQA1* | 01:01 | -DQB1* | 05:01 | 1 |
| HLA- C* | 04:01 | -B* | 35:08 | -DRB1* | 16:02 | -DQA1* | 01:02 | -DQB1* | 05:02 | 1 | HLA- C* | 15:13 | -B* | 51:01 | -DRB1* | 04:02 | -DQA1* | 01:01 | -DQB1* | 03:02 | 1 |
| HLA- C* | 04:01 | -B* | 50:01 | -DRB1* | 03:01 | -DQA1* | 05:01 | -DQB1* | 02:01 | 1 | HLA- C* | 15:13 | -B* | 51:01 | -DRB1* | 04:02 | -DQA1* | 03:01 | -DQB1* | 03:02 | 1 |
| HLA- C* | 04:01 | -B* | 51:01 | -DRB1* | 04:02 | -DQA1* | 03:01 | -DQB1* | 03:02 | 1 | HLA- C* | 16:01 | -B* | 44:03 | -DRB1* | 07:01 | -DQA1* | 02:01 | -DQB1* | 02:02 | 1 |
| HLA- C* | 04:01 | -B* | 51:01 | -DRB1* | 15:06 | -DQA1* | 01:05 | -DQB1* | 05:02 | 1 | HLA- C* | 16:01 | -B* | 45:01 | -DRB1* | 01:02 | -DQA1* | 01:01 | -DQB1* | 05:01 | 1 |
| HLA- C* | 04:03 | -B* | 13:01 | -DRB1* | 16:02 | -DQA1* | 01:02 | -DQB1* | 05:02 | 1 | HLA- C* | 16:02 | -B* | 51:01 | -DRB1* | 03:01 | -DQA1* | 05:01 | -DQB1* | 02:01 | 1 |
| HLA- C* | 06:02 | -B* | 13:02 | -DRB1* | 01:02 | -DQA1* | 03:01 | -DQB1* | 05:01 | 1 | HLA- C* | 16:02 | -B* | 51:01 | -DRB1* | 13:02 | -DQA1* | 01:02 | -DQB1* | 06:04 | 1 |
| HLA- C* | 06:02 | -B* | 37:01 | -DRB1* | 04:03 | -DQA1* | 03:01 | -DQB1* | 03:02 | 1 | HLA- C* | 16:02 | -B* | 51:08 | -DRB1* | 13:03 | -DQA1* | 05:05 | -DQB1* | 03:01 | 1 |
| HLA- C* | 06:02 | -B* | 41:01 | -DRB1* | 07:01 | -DQA1* | 02:01 | -DQB1* | 02:02 | 1 | HLA- C* | 16:04 | -B* | 44:02 | -DRB1* | 04:04 | -DQA1* | 03:01 | -DQB1* | 03:02 | 1 |
| HLA- C* | 06:02 | -B* | 45:01 | -DRB1* | 09:01 | -DQA1* | 03:03 | -DQB1* | 02:01 | 1 | HLA- C* | 17:01 | -B* | 41:01 | -DRB1* | 01:02 | -DQA1* | 01:01 | -DQB1* | 05:01 | 1 |
| HLA- C* | 06:02 | -B* | 50:01 | -DRB1* | 04:02 | -DQA1* | 03:01 | -DQB1* | 03:02 | 1 | HLA- C* | 17:01 | -B* | 42:01 | -DRB1* | 03:02 | -DQA1* | 04:01 | -DQB1* | 04:02 | 1 |
| HLA- C* | 06:02 | -B* | 50:01 | -DRB1* | 07:01 | -DQA1* | 01:02 | -DQB1* | 02:02 | 1 | HLA- C* | 17:01 | -B* | 42:01 | -DRB1* | 03:07 | -DQA1* | 01:02 | -DQB1* | 05:02 | 1 |
| HLA- C* | 06:02 | -B* | 50:01 | -DRB1* | 07:01 | -DQA1* | 02:01 | -DQB1* | 02:01 | 1 | HLA- C* | 17:01 | -B* | 42:01 | -DRB1* | 11:02 | -DQA1* | 05:05 | -DQB1* | 03:19 | 1 |
| HLA- C* | 06:02 | -B* | 53:01 | -DRB1* | 04:02 | -DQA1* | 03:01 | -DQB1* | 03:02 | 1 | HLA- C* | 17:01 | -B* | 42:01 | -DRB1* | 15:03 | -DQA1* | 01:02 | -DQB1* | 06:02 | 1 |
| HLA- C* | 06:02 | -B* | 57:01 | -DRB1* | 07:01 | -DQA1* | 02:01 | -DQB1* | 04:02 | 1 | HLA- C* | 18:01 | -B* | 81:01 | -DRB1* | 11:01 | -DQA1* | 05:05 | -DQB1* | 02:01 | 1 |

Table S 9: Names for Conserved Extended Haplotypes (CEH) observed in the current study. ^‡^Proposed.

| CEH | HLA-C | HLA-B | HLA-DRB1 | HLA-DQA1 | HLA-DQB1 | Reference |
| --- | --- | --- | --- | --- | --- | --- |
| 8.1 | 07:01 | 08:01 | 03:01 | 05:01 | 02:01 | ^25-28^ |
| 8.2 | 07:02 | 08:01 | 03:01 | 05:01 | 02:01 | ^29-31^ |
| 60.4^‡^ | 15:02 | 40:06 | 16:02 | 01:02 | 05:02 | Consensus from UAE cohort |
| 51.2^‡^ | 16:02 | 51:01 | 16:01 | 01:02 | 05:02 | Consensus from UAE cohort |
| 58.1 | 03:02 | 58:01 | 03:01 | 05:01 | 02:01 | ^32^ |
| 58.2^‡^ | 03:02 | 58:01 | 16:01 | 01:02 | 05:02 | Consensus from UAE cohort |

**References**

1 Gonzalez-Galarza, F. F. *et al.* Allele frequency net database (AFND) 2020 update: gold-standard data classification, open access genotype data and new query tools. *Nucleic acids research* **48**, D783-d788, doi:10.1093/nar/gkz1029 (2020).

2 Saito, S., Ota, S., Yamada, E., Inoko, H. & Ota, M. Allele frequencies and haplotypic associations defined by allelic DNA typing at HLA class I and class II loci in the Japanese population. *Tissue antigens* **56**, 522-529, doi:10.1034/j.1399-0039.2000.560606.x (2000).

3 Torimiro, J. N. *et al.* HLA class I diversity among rural rainforest inhabitants in Cameroon: identification of A*2612-B*4407 haplotype. *Tissue antigens* **67**, 30-37, doi:10.1111/j.1399-0039.2005.00527.x (2006).

4 Zúñiga, J. *et al.* HLA Class I and Class II Conserved Extended Haplotypes and Their Fragments or Blocks in Mexicans: Implications for the Study of Genetic Diversity in Admixed Populations. *PLOS ONE* **8**, e74442, doi:10.1371/journal.pone.0074442 (2013).

5 Zou, J., Shen, G., Qiang, W., Zhu, Y. Y. & Li, W. X. Study on the polymorphisms of HLA-ABCDQB1DRB1 alleles and haplotypes in Hubei Han population of China. *International journal of immunogenetics* **48**, 8-15, doi:10.1111/iji.12516 (2021).

6 Páez-Gutiérrez, I. A., Hernández-Mejía, D. G., Vanegas, D., Camacho-Rodríguez, B. & Perdomo-Arciniegas, A. M. HLA-A, -B, -C, -DRB1 and -DQB1 allele and haplotype frequencies of 1463 umbilical cord blood units typed in high resolution from Bogotá, Colombia. *Human immunology* **80**, 425-426, doi:10.1016/j.humimm.2019.03.006 (2019).

7 Tan, L. K. *et al.* HLA-A, -B, -C, -DRB1 and -DQB1 alleles and haplotypes in 951 Southeast Asia Malays from Peninsular Malaysia. *Human immunology* **77**, 818-819, doi:10.1016/j.humimm.2016.06.022 (2016).

8 Arrieta-Bolaños, E. *et al.* High-resolution HLA allele and haplotype frequencies in majority and minority populations of Costa Rica and Nicaragua: Differential admixture proportions in neighboring countries. *Hla* **91**, 514-529, doi:10.1111/tan.13280 (2018).

9 Grubic, Z. *et al.* HLA-A, HLA-B and HLA-DRB1 allele and haplotype diversity among volunteer bone marrow donors from Croatia. *International journal of immunogenetics* **41**, 211-221, doi:10.1111/iji.12117 (2014).

10 Mohyuddin, A. *et al.* HLA polymorphism in six ethnic groups from Pakistan. *Tissue antigens* **59**, 492-501, doi:10.1034/j.1399-0039.2002.590606.x (2002).

11 Llanes, A. *et al.* HLA allele and haplotype frequencies in the Panamanian population. *Human immunology* **82**, 5-7, doi:10.1016/j.humimm.2020.11.006 (2021).

12 Norman, P. J. *et al.* Co-evolution of human leukocyte antigen (HLA) class I ligands with killer-cell immunoglobulin-like receptors (KIR) in a genetically diverse population of sub-Saharan Africans. *PLoS genetics* **9**, e1003938, doi:10.1371/journal.pgen.1003938 (2013).

13 Schmidt, A. H. *et al.* High-resolution human leukocyte antigen allele and haplotype frequencies of the Polish population based on 20,653 stem cell donors. *Human immunology* **72**, 558-565, doi:10.1016/j.humimm.2011.03.010 (2011).

14 Seshasubramanian, V. *et al.* Malayalam speaking population from South India: Common five-locus haplotypes in Malayalam speaking population. *Hla* **92**, 432-434, doi:10.1111/tan.13389 (2018).

15 Rajalingam, R. *et al.* Distinctive KIR and HLA diversity in a panel of north Indian Hindus. *Immunogenetics* **53**, 1009-1019, doi:10.1007/s00251-001-0425-5 (2002).

16 Huh, J. Y. *et al.* HLA-A, -B and -DRB1 polymorphism in Koreans defined by sequence-based typing of 4128 cord blood units. *International journal of immunogenetics* **40**, 515-523, doi:10.1111/iji.12067 (2013).

17 Narayan, S. *et al.* Human leucocyte antigen (HLA)-A, -B, -C, -DRB1 and -DQB1 haplotype frequencies from 2491 cord blood units from Tamil speaking population from Tamil Nadu, India. *Molecular biology reports* **45**, 2821-2829, doi:10.1007/s11033-018-4382-6 (2018).

18 Lee, K. W., Oh, D. H., Lee, C. & Yang, S. Y. Allelic and haplotypic diversity of HLA-A, -B, -C, -DRB1, and -DQB1 genes in the Korean population. *Tissue antigens* **65**, 437-447, doi:10.1111/j.1399-0039.2005.00386.x (2005).

19 Enrich, E. *et al.* HLA-A, -B, -C, -DRB1, and -DQB1 allele and haplotype frequencies: An analysis of umbilical cord blood units at the Barcelona Cord Blood Bank. *Hla* **94**, 347-359, doi:10.1111/tan.13644 (2019).

20 Elamin, N. E. *et al.* Identification of four new HLA-Cw alleles in the Sudanese population. *Tissue antigens* **69**, 270-272, doi:10.1111/j.1399-0039.2006.00789.x (2007).

21 Williams, F. *et al.* High resolution HLA-DRB1 identification of a Caucasian population. *Human immunology* **65**, 66-77, doi:10.1016/j.humimm.2003.10.004 (2004).

22 Hajjej, A., Almawi, W. Y., Hattab, L., El-Gaaied, A. & Hmida, S. The investigation of the origin of Southern Tunisians using HLA genes. *Journal of Human Genetics* **62**, 419-429, doi:10.1038/jhg.2016.146 (2017).

23 Rendine, S. *et al.* Estimation of human leukocyte antigen class I and class II high-resolution allele and haplotype frequencies in the Italian population and comparison with other European populations. *Human immunology* **73**, 399-404, doi:10.1016/j.humimm.2012.01.005 (2012).

24 Johansson, A., Ingman, M., Mack, S. J., Erlich, H. & Gyllensten, U. Genetic origin of the Swedish Sami inferred from HLA class I and class II allele frequencies. *European journal of human genetics : EJHG* **16**, 1341-1349, doi:10.1038/ejhg.2008.88 (2008).

25 Dorak, M. T. *et al.* Conserved extended haplotypes of the major histocompatibility complex: further characterization. *Genes and immunity* **7**, 450-467, doi:10.1038/sj.gene.6364315 (2006).

26 Degli-Esposti, M. A. *et al.* Ancestral haplotypes: conserved population MHC haplotypes. *Human immunology* **34**, 242-252, doi:10.1016/0198-8859(92)90023-g (1992).

27 Degli-Esposti, M. A. *et al.* Ancestral haplotypes reveal the role of the central MHC in the immunogenetics of IDDM. *Immunogenetics* **36**, 345-356, doi:10.1007/bf00218041 (1992).

28 Yunis, E. J. *et al.* Inheritable variable sizes of DNA stretches in the human MHC: conserved extended haplotypes and their fragments or blocks. *Tissue antigens* **62**, 1-20, doi:https://doi.org/10.1034/j.1399-0039.2003.00098.x (2003).

29 Kaur, G. *et al.* Autoimmune-associated HLA-B8-DR3 haplotypes in Asian Indians are unique in C4 complement gene copy numbers and HSP-2 1267A/G. *Human immunology* **69**, 580-587, doi:10.1016/j.humimm.2008.06.007 (2008).

30 Mehra, N. K., Kumar, N., Kaur, G., Kanga, U. & Tandon, N. Biomarkers of susceptibility to type 1 diabetes with special reference to the Indian population. *J Indian Journal of Medical Research* **125**, 321-344 (2007).

31 Witt, C. S. *et al.* Common HLA-B8-DR3 haplotype in Northern India is different from that found in Europe. *Tissue antigens* **60**, 474-480, doi:10.1034/j.1399-0039.2002.600602.x (2002).

32 Cheong, K. Y. *et al.* Localization of central MHC genes influencing type I diabetes. *Human immunology* **62**, 1363-1370, doi:10.1016/s0198-8859(01)00351-2 (2001).
