# Supplementary material for: Characterizing the diversity of MHC conserved extended haplotypes using families from the United Arab Emirates
Source: Sci Rep. 2022 May 3;12:7165. doi: 10.1038/s41598-022-11256-y (PMC9065074; doi:10.1038/s41598-022-11256-y)
Supplement: Supplementary file 2 — Supplementary Figures. [file 41598_2022_11256_MOESM2_ESM.docx]

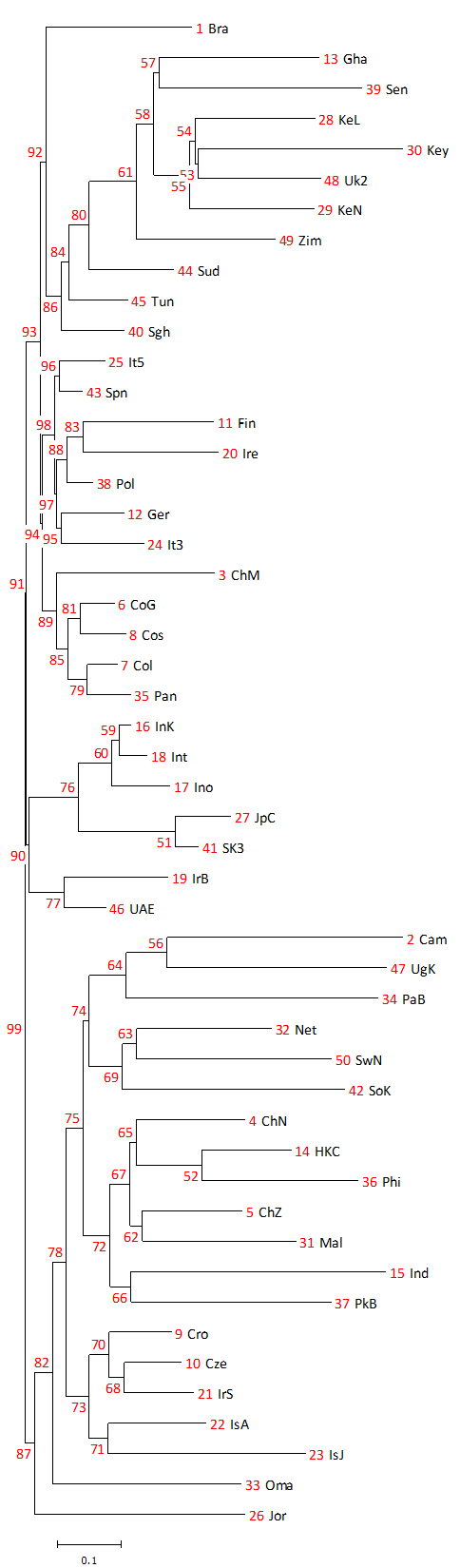


Figure S1 Complete neighbor-joining phylogenetic tree showing relatedness between the current UAE cohort and 49 other populations calculated using frequencies of HLA-A, -B and -DRB1 loci. For the complete list of abbreviations and descriptions of datasets used, refer to Table S 7.
